# An early effect of the parafoveal preview on post-saccadic processing of English words

**DOI:** 10.3758/s13414-024-02916-4

**Published:** 2024-07-02

**Authors:** David Melcher, Ani Alaberkyan, Chrysi Anastasaki, Xiaoyi Liu, Michele Deodato, Gianluca Marsicano, Diogo Almeida

**Affiliations:** 1 Psychology Program, Division of Science, New York University Abu Dhabi, PO Box 129188, Abu Dhabi, United Arab Emirates; 2 Center for Brain and Health, NYUAD Research Institute, New York University Abu Dhabi, PO Box 129188, Abu Dhabi, United Arab Emirates; 3 Department of Psychology, Princeton University, Washington Rd, Princeton, NJ 08540 USA; 4 Department of Psychology, University of Bologna, Viale Berti Pichat 5, 40121 Bologna, Italy; 5 Centre for Studies and Research in Cognitive Neuroscience, University of Bologna, Via Rasi e Spinelli 176, 47023 Cesena, Italy

**Keywords:** Eye movements and reading, Electrophysiology, Methods: VEP, EEG, MEG

## Abstract

A key aspect of efficient visual processing is to use current and previous information to make predictions about what we will see next. In natural viewing, and when looking at words, there is typically an indication of forthcoming visual information from extrafoveal areas of the visual field before we make an eye movement to an object or word of interest. This “preview effect” has been studied for many years in the word reading literature and, more recently, in object perception. Here, we integrated methods from word recognition and object perception to investigate the timing of the preview on neural measures of word recognition. Through a combined use of EEG and eye-tracking, a group of multilingual participants took part in a gaze-contingent, single-shot saccade experiment in which words appeared in their parafoveal visual field. In valid preview trials, the same word was presented during the preview and after the saccade, while in the invalid condition, the saccade target was a number string that turned into a word during the saccade. As hypothesized, the valid preview greatly reduced the fixation-related evoked response. Interestingly, multivariate decoding analyses revealed much earlier preview effects than previously reported for words, and individual decoding performance correlated with participant reading scores. These results demonstrate that a parafoveal preview can influence relatively early aspects of post-saccadic word processing and help to resolve some discrepancies between the word and object literatures.

## Introduction

Natural viewing, whether looking around a scene or reading words, involves a close interaction between the visual system and the oculomotor system. Typically, we use information from outside the fovea (extrafoveal vision) to identify objects or words of interest and then direct our gaze there so that it can be processed at the fovea, where acuity and color vision are the best. Natural viewing is made up of a cycle: foveal processing, extrafoveal processing to identify the next target of the saccade, the saccade, and then foveal processing again. In contrast, most research involves stable eye fixation and the sudden appearance, and then disappearance, of a stimulus. This raises the question of how object and word processing are influenced by a parafoveal preview.

During each fixation, it is well known that we recognize the object or word in the fovea (Sereno & Rayner, 2003). This process is rapid and supports efficient comprehension by facilitating transmission of visual information from the retina to the visual cortex, word/object identification, and programming of the next eye movement (Degno & Liversedge, 2020). While foveal processing is clearly important and well studied experimentally, a number of studies have shown that we also utilize information from outside the fovea (extrafoveal, typically parafoveal information within 5° of visual angle) to preview upcoming objects or words before making a saccade to fixate on them directly (Baccino, 2011; Degno & Liversedge, 2020). In theory, this parafoveal information can be used not only to guide the saccade landing position but also to reduce the number of possible objects or words that will soon be recognized. In contrast to a laboratory experiment in which a single object or word is suddenly presented on the screen, in which the item could potentially be any word or object, while during natural viewing the visual system has access to information to reduce this possible space of potential items, even prior to foveating the item**.** Thus, the parafoveal preview dramatically reduces the uncertainty regarding what might be present on the fovea after the saccade.

This potential interaction between parafoveal and subsequent foveal processing has traditionally been studied in two parallel literatures: object and scene perception on the one hand and word recognition on the other. In the object perception literature, a large number of studies have looked at how pre-saccadic information (the “preview”) influences post-saccadic processing of the visual stimulus after the saccade lands on the target. Such studies indicate that visual motion information (Fracasso et al., 2010; Melcher & Morrone, 2003), shape/identity (Cha & Chong, 2014; Demeyer et al., 2009; Ganmor et al., 2015; Gordon et al., 2008; Harrison & Bex, 2014; Herwig, 2015; Melcher, 2007; Wijdenes et al., 2015; Paeye et al., 2017; Pertzov et al., 2009; Prime et al., 2006, 2011; Van Eccelpoel et al., 2008; Zimmermann et al., 2013; Wolfe & Whitney, 2015) and even color (Eymond et al., 2019; Wittenberg et al., 2008) from the pre-saccadic peripheral preview can influence post-saccadic processing. In the case of face images, for example, the preview face can subtly change the reported percept of the post-saccadic face (Melcher, 2005; Wolfe & Whitney, 2015) and makes participants faster and more accurate at making perceptual judgments about that face after the saccade (for review, see Huber-Huber et al., 2021).

Many of the issues studied in the temporal integration of visual object information across saccades mirror those investigated in masked word priming, in which a prime word is presented for very brief durations (< 60 ms) and is immediately substituted by the target, which acts as the backwards mask of the prime word (Forster, 1998; Forster et al., 2003, for review). Despite important differences (in masked priming, the stimuli are presented foveally, as opposed to first parafoveally and then foveally), similar conclusions to trans-saccadic object recognition studies are often reported. For example, similarly to valid preview conditions with objects, full repetition of words (but not pseudowords or purely orthographically similar words), elicit shorter response times (RTs) and more accurate lexical decisions (Forster & Davis, 1984). As in studies of trans-saccadic object perception, described above, there is a debate regarding to which extent the visual system extracts more low-level or high-level information (Forster, 1998; Perea & Gotor, 1997).

Beyond single words, in the case of sentence reading, studies employing the preview paradigm have reported that post-saccadic word processing is typically more efficient in the valid preview condition compared to when there is no/reduced preview or an invalid preview. One useful method is the gaze-contingent preview paradigm (Rayner, 1998). In this paradigm, participants are typically instructed to freely read the words while their gaze position is tracked. A predetermined target object or word is manipulated in a gaze-contingent manner. In one version of this paradigm, the target can change identity during the saccade, such that the pre-saccadic parafoveal input differs from the post-saccadic foveal image (an “invalid” condition). In other versions, the quality of the parafoveal information is manipulated, such as by blurring or replacing the letters in words outside of the fovea with symbols (e.g., #####), to create a sort of “tunnel vision” (reduced or neutral condition). In both cases, the nature of preview effects is then estimated by comparing the valid (normal) viewing condition with an "invalid" preview condition (the stimulus changes across the saccade) or a “reduced”/neutral preview condition (Blanchard et al., 1989; Chang et al., 2020; Hohenstein & Kliegl, 2014; Li et al., 2015; Pan et al., 2020; Schotter & Fennell, 2019; Schotter et al., 2012). Therefore, it is evident that information is acquired from the parafoveal region and integrated with the subsequently foveated visual information (Inhoff & Rayner, 1986). At the same time, sentence reading involves the construction of complex linguistic structures over multiple saccades across a time period of seconds, which is quite different from the object and word recognition studies mentioned above. Nevertheless, the existence of preview effects aligns with the notion that the sensory and oculomotor systems are constantly engaged in predicting imminent foveal input. These findings highlight the dynamic interplay between visual and motor processes, emphasizing the role of trans-saccadic processing in facilitating efficient and anticipatory visual perception.

### Co-registration of combined eye-tracking and EEG: Fixation-related potentials

In recent years, the joint consideration of eye movements and electroencephalography (EEG) has granted new perspectives to the study of active vision (Dimigen et al., 2012). Unlike the traditional event-related potential (ERP) paradigm, which relies on externally triggered stimulus presentation, the co-registration of eye tracking and EEG allows the study of “fixation-related potentials” (FRPs), a distinct category of ERPs that are aligned to eye movements. By tracking a participant's eye movements, the FRP approach enables researchers to establish the EEG signal when a subject is fixating specific elements, such as objects or words. This allows for the precise alignment of cognitive processes with electrophysiological measurements, capturing the neural activity associated with specific fixations (Hutzler et al., 2007).

Thus, a more nuanced understanding of the preview effect can be achieved with effective eye-tracking and EEG co-registration (EYE-EEG) methodologies and data analytic techniques (for review: Huber-Huber et al., 2021; Nikolaev et al., 2016). In such studies, one or more stimuli is presented outside the fovea while the participant maintains fixation. Then, at some point, the participant is free to look at one of the stimuli. As in the gaze-contingent reading paradigms, the validity of the extrafoveal (sometimes beyond 5°, so not strictly parafoveal) preview can be manipulated by, for example, changing some feature of the stimulus during the saccade or by reducing the visibility of the extrafoveal preview (Liu et al., 2022; Liu et al., 2023). One set of studies has shown that the nature of the preview stimulus affects the face-sensitive N170 component (Buonocore et al, 2020; De Lissa, et al., 2019; Huber-Huber et al., 2019). In particular, the fixation-related N170 was greatly reduced in the valid, compared to neutral or invalid, preview conditions (in which faces were scrambled or presented upside-down).

Eye-tracking and EEG have been used together to make inferences about the underlying cognitive processes of parafoveal processing, whether in the case of visual objects, scenes, words, or even sentences (for extensive reviews, see Degno & Liversedge, 2020; Huber-Huber et al., 2021). There are several advantages in using EEG to provide millisecond-by-millisecond temporal resolution estimates of the neural activity of the brain and linking it to eye-tracking to better understand neural processing related to fixations and saccades (Dimigen et al., 2011; Fabius et al., 2023). Using EEG, researchers can capture ERPs, which are time-locked brain responses related to specific cognitive events (Luck, 2005). For example, the N170 component is known to be sensitive to visual and orthographic processing, including the recognition of faces and words. Likewise, a number of studies have investigated the nature of the N400 component in word processing (Kutas & Federmeier, 2011). There is a long history of investigating the link between specific ERP components and specific stages of sensory and cognitive processing (for review, see Almeida & Poeppel, 2013; Carreiras et al., 2014; Grainger & Holcomb, 2009; Sprouse & Almeida, 2023).

In the context of the preview effect, of particular interest here is the N170, which has been studied to investigate the influence of parafoveal preview on both face and word recognition. Studies have shown that the N170 amplitude can be modulated by the quality or congruency of the parafoveal preview. In the case of faces, presenting a valid face preview prior to the saccade leads to a dramatic reduction of the N170 compared to when a face “appears” upon the new fixation. Also in the case of words, when the preview word matches the target word, resulting in a valid preview, the N170 response is often reduced compared to invalid previews (Degno et al., 2019a, 2019b; Dimigen et al., 2012; Kornrumpf et al., 2016; Niefind & Dimigen, 2016). This reduction in N170 amplitude has been interpreted in terms of more efficient visual and orthographic processing, suggesting facilitated word recognition.

At the same time, it is important to note that ERP components cannot easily be linked to a single cognitive process, and that the evidence for actual lexical, as opposed to visual and orthographic processing in the N170 time-window is equivocal (Compton et al., 1991; Cornelissen et al., 2003; Maurer et al., 2005). This is particularly critical in the case of natural reading, where word recognition is only one of the many cognitive operations happening in parallel to support language understanding. For example, the N170 has been observed in both language-related and face recognition related processing (Huber-Huber et al., 2021; Kornrumpf et al., 2016). Thus, the interpretation of the implication of ERPs in word recognition is challenging given the many complex processes involved in reading (Picton et al., 2000).

Both words and faces are considered special stimuli, with some commonalities in terms of being processed holistically and with similar, potentially overlapping, brain regions involved in the two tasks, with evidence for different lateralizations in symmetric areas of the right (face) and left (word) hemispheres (Burns & Bukach, 2021; Feizabadi et al., 2021; Robinson et al., 2017). In terms of the two literatures (objects and words), perhaps the largest discrepancy has to do with the timing of preview effects (for discussion of this issue, see Huber-Huber et al., 2021). A number of visual object studies have suggested that information about the preview stimulus is present in the EEG signal well before saccade onset and interacts with post-saccadic processing of the target within a time period less than 100 ms (for review, see Huber-Huber et al., 2021). This estimate is dramatically shorter than what would be inferred from previous studies using word stimuli. However, many of the studies with objects have used multivariate approaches that rely on high signal-to-noise in order to show sensitivity to small differences between conditions. Such sensitivity would not, in principle, be present when many different stimuli (such as several objects or a sentence full of words) are active in the brain all at the same time since EEG/MEG sensors combine these separate types of information due to volume conductance. Previous studies have combined EEG and eye-tracking to measured fixated-related responses to words that are embedded in sentences, or within lists of words (Dimigen et al., 2012; Kornrumpf et al., 2016; Niefind & Dimigen, 2016) as well as word pairs (e.g., Baccino & Manunta, 2005; López-Peréz et al., 2016; Simola et al., 2009). Given that EEG correlates of word processing continue over many hundreds of milliseconds, the parafoveal preview is being processed at the same time that information about the currently fixated item is still present in the EEG signal. Similarly, the processing of the previous word contaminates the EEG signal at the onset of the new target fixation. Studies of word pairs suggest that the parafovea preview and foveal stimuli are processed in parallel and that the preview effect can overlap with parafovea-on-fovea effects (López-Peréz et al., 2016). This makes it difficult to disentangle the timing of such effects and to make any strong claims about the timing of the preview effect either in the pre-saccadic period or the post-saccadic fixation on the target of interest.

One novel method to minimize the averaging of signals from different objects or words has been to use frequency tagging (flashing the stimuli at different rates) to measure the time course of processing of different words (Pan et al., 2021, 2023). For example, Pan and colleagues found that a parafoveal target evoked a stronger frequency tagging response during pre-target fixation when it was followed by a word with low lexical frequency (as opposed to a more common word). In addition, they demonstrated that the size of the lexical parafoveal processing effect was correlated with individual reading speed. By tagging different words at different flashing frequencies, those authors were able to overcome the issue of how to separate neural signals related to the many different processes happening simultaneously when looking around a complex scene or set of words.

Another approach is to start by looking at preview effects for only one or two objects or words present on the screen at a time. One advantage of the single-shot saccade paradigm used previously with objects is that, like frequency tagging, it isolates the effects of the extrafoveal preview from other aspects of real-world scene viewing and sentence reading that should also influence decoding and, more generally, the fixation-related response. In general, visual processing of objects or words is spatially and temporally distributed, meaning that when we process multiple items their relative signals overlap in the EEG/MEG signal (for review, see Robinson et al., 2019). Separating stimuli in time increases the multivariate decodability of object representations (Grootswagers et al., 2019; Mohsenzadeh et al., 2018).

Of particular interest for the current study, Kornrumpf and colleagues (Kornrumpf et al., 2016) compared the influence of the preview effect during natural reading with a “passive reading” condition in which the participant maintained fixation but the words themselves moved across the screen so that each words was shown sequentially at the fovea, mimicking reading. In the case of natural reading, there was a strong preview effect at around 200–300 ms. Specifically, the evoked response was greatly reduced when there was a valid preview, and moreover, the extent of this reduction varied as a function of the amount of useful information available in the preview (such as the number of visible letters in the preview). This raises the question of whether a similar reduction in the fixation-related potentials will be found in a single-shot saccade paradigm.

One goal of the current study was to examine the preview effect for recognition of English words in a simplified context in which both univariate and multivariate EEG analyses are appropriate. By using a nearly identical paradigm to that used previously with faces, we can directly compare the FRPs and the multivariate decoding results for the two stimulus types, albeit with different participants. This allows us to investigate this seeming discrepancy in terms of the timing of word preview and face preview effects. While preview effects have been studied for a wide range of visual stimulus types, as described above, the comparison between words and faces is particularly relevant in terms of the timing and relative location in the brain of specialized neural processing.

At the same time, however, there is the concern that removing the reading context and focusing on just single words might also alter the preview effect. As stated above, sentence reading is more complex than just word recognition. This sentence context can influence eye movement measures, such as word skipping and initial fixation durations, as well as ERPs/FRPs (Antúnez et al., 2022; Schotter, 2013; Schotter & Jia, 2016; Schotter et al., 2015; Veldre & Andrews, 2016; for review, see Andrews & Veldre, 2019; Schotter et al., 2018).

### The present study

In our experiment, we used co-registration of eye-tracking and EEG using a single word, one-saccade, gaze-contingent paradigm to explore the role of the preview on word recognition. Considering that other studies involving faces or reading tasks consistently report a preview effect (Dimigen et al., 2011; Schotter et al., 2012), we hypothesized that we would find a robust preview effect with our pared-down design with single words. This would allow for a more direct comparison of preview effects for words and faces and the seeming differences in the timing of these effects. We manipulated the parafoveal preview of the upcoming word to be either valid (same as the actual word) or neutral/invalid (preview was just a number string). In terms of terminology, in this manuscript we will refer to this as “invalid” since we used number characters that were not the same as the word target, as opposed to a set of symbols (such as #) as commonly used ina “neutral” condition. A single preview stimulus was presented on each trial on either the left or the right side of fixation. Following the presentation of the saccade target, once the saccade was detected we presented a word stimulus. Participants were instructed to read the word, before it disappeared from the screen, in order to then make a same/different judgment for a subsequently presented word, which was identical to that word on 50% of trials. We hypothesized that the preview effect would greatly reduce the amplitude of the FRP at the electrodes of interest compared to the invalid preview. In particular, we looked to see whether there would be a similar validity effect at around 200–300 ms as found in the Kornrumpf and colleagues’ study (Kornrumpf et al., 2016). If so, then this would suggest that our single-saccade paradigm could be used to meaningfully explore the nature of preview effects on word recognition.

While words are often viewed in isolation and there is large literature focusing on recognition of single words, it is also clear that processing of words can be influenced by their context such as when in phrases or sentences. This raises the question of why to study single word recognition here, especially in the context of preview effects. As described above, a major motivation is to compare word processing with faces and other objects, which is a topic of much research (for recent review examples, see Behrmann & Plaut, 2020; Feizabadi et al., 2021; Robotham & Starrfelt, 2017; Roission & Lochy, 2022).

In addition, however, there are a number of potential advantages of a more simple, single-shot version of studying the parafoveal preview based on the ability to precisely control what information is available as part of the parafoveal preview and for how long. First, fixation durations during natural viewing vary in duration and, indeed, such fixation durations are often used as a dependent variable in studies of scene or text viewing. The single saccade paradigm used here offers experimental control and ensures that any lack of a preview effect is not due to the participant making a saccade too early, before they have fully processed all potentially available parafoveal information. Second, the location of the stimulus in terms of retinal eccentricity is controlled, which is important given that the quality of the preview is influenced by its visibility and the presence of other stimuli can create crowding which further limits the parafoveal preview utility (Chiu & Drieghe, 2023; Frömer et al., 2015). In terms of co-registration of EEG and saccades, it is well known that the size of the saccade influences the oculomotor artifact.

Moreover, the use of single words allows us to control what other word-related information is being processed by the participant. The neural signals being averaged by the EEG electrodes come from all of the activity within a relatively large area of the brain (inversely to the distance from the electrodes), mixing signals from different processes. Here we report the results using single words, but in other ongoing studies we have introduced a hybrid version which presents additional words, either visually (at fixation) or auditorily (through speakers) in order to add onto these single-word parafoveal preview effects.

Because we used only a single word, we have implemented both univariate (evoked potentials) and multivariate (decoding) approaches to track the processing of the preview and the post-saccadic stimulus over time. As described above, decoding sensitivity is higher when there are no competing stimuli. A number of studies using non-word stimuli have reported relatively early (<100 ms) effects of the preview (for discussion of this issue, see Huber-Huber et al., 2021), presumably reflecting more low-level feature analysis, while many studies of words have looked at much “later” effects. The single word approach allowed us to maximize the sensitivity of multivariate analyses to the manipulation of interest without the added noise of having multiple stimuli present in the visual field in space and over time.

In many previous boundary-paradigm studies, the target word is changed during the saccade. Thus, the post-saccadic fixation-evoked response measured by co-registered EEG and eyetracking is likely a combination of responses, including the desired “lack of valid preview” effect, but also a prediction violation response. In the current study, we use a number string as the invalid preview condition. However, the target was always a word. Thus, the invalid condition failed to license any useful predictive information about the potential identity of the post-saccadic target. Our aim was to isolate, at least in part, the positive preview benefit from a specific prediction violation response.

Also of interest and note in the present study was that we measured the preview effect in a highly diverse participant group that was bilingual/multilingual. Almost all of our participants acquired English as a second language, typically around 5 years of age. By including a more diverse and multilingual participant group, this study allows for a comparison of our findings with previous studies using a more homogenous, first language (L1) study population. Given the high prevalence of bilingualism and multilingualism in the world, this study contributes to moving beyond monolingual and “WEIRD'' (Western, Educated, Industrialized, Rich, and Democratic) research populations in psychology research (Henrich et al., 2010; Kroll & Dussias, 2017).

## Method

### Participants

Concurrent EEG and eye-tracking data were recorded from 20 participants (13 female) who were recruited using the SONA participants pool within the Psychology Department at New York University Abu Dhabi (NYUAD). Written informed consent was obtained prior to the experiment in line with the Declaration of Helsinki and the participants received monetary reimbursement or course credit for their efforts. The study was approved by the Institutional Review Board (IRB) at NYUAD. All participants reported normal or corrected-to normal vision. Sixteen of them were right-handed, and 18 were right-eye dominant. The mean age was 22 years, ranging from 19 to 29 years. All participants were bi/multilingual, fluent in English and at least another language (e.g., Hindi, Urdu, and Malayalam). Three participants were excluded from the data analysis because of a failure to synchronize the eye-tracking and EEG data. One participant was excluded as it was not possible to obtain a proper amount of events during the epoching stage of their data.

Language ability was measured through a self-report questionnaire (LEAP-Q: Marian et al., 2007), with the results summarized in Table 1. This included participants who reported English as their dominant, second-most dominant, and even third-most dominant language.

**Table 1:** Self-reported language history and proficiency for participants from the Language Experience and Proficiency Questionnaire (LEAP-Q) across English dominance groups

	English 1st dominant			English 2nd dominant			English 3rd dominant		
	(8 participants)			(10 participants)			(2 participants)		
Language history measures	M	Var	SD	M	Var	SD	M	Var	SD
Self-Reported^1^ Proficiency									
Understanding	9.13	0.35	0.13	8.20	1.07	1.03	7.50	0.50	0.71
Speaking	8.63	0.52	0.27	8.30	0.68	0.82	7.00	2.00	1.41
Reading	8.75	0.71	0.50	8.80	1.51	1.23	9.00	0.00	0.00
Age Milestones (years)									
Started learning	4.25	3.36	1.83	8.50	49.83	7.06	4.00	2.00	1.41
Attained fluency	8.63	8.84	2.97	10.56	14.28	3.78	16.00	0.00	0.00
Started reading	5.38	3.41	1.85	6.89	9.11	3.02	4.00	2.00	1.41
Attained reading fluency	9.75	14.21	3.77	10.67	22.50	4.74	8.00	0.00	0.00
Immersion duration (years)									
In a country	9.88	45.55	6.75	7.40	37.16	6.10	1.00	0.00	0.00
In a school	9.00	92.00	9.59	4.10	49.21	7.02	2.50	12.50	3.54
In a family	13.50	23.14	4.81	10.10	43.66	6.61	9.50	0.50	0.71
Contributions to language learning (percent)									
From family	7.50	10.00	3.16	5.90	7.43	2.73	5.00	8.00	2.83
From friends	2.86	10.48	3.24	3.56	12.03	3.47	2.00	0.00	0.00
From reading	8.13	2.13	1.46	9.00	1.78	1.33	7.50	4.50	2.12
From language apps/websites	3.20	19.70	4.44	6.38	13.41	3.66	6.50	0.50	0.71
From TV	8.13	2.98	1.73	8.20	1.73	1.32	8.50	4.50	2.12
From radio	5.25	8.79	2.96	7.33	4.75	2.18	4.00	32.00	5.66
From education	8.38	9.13	3.02	8.80	5.73	2.39	9.50	0.50	0.71
Extent of language exposure (percent)									
Friends	7.88	8.98	3.00	7.80	9.73	3.12	7.00	8.00	2.83
Family	3.83	8.57	2.93	5.50	11.90	3.45	1.00	0.00	0.00
Reading	8.50	8.00	2.83	9.40	0.93	0.97	8.00	8.00	2.83
Language apps/websites	5.67	17.33	4.16	5.00	21.00	4.58	4.50	4.50	2.12
TV	7.75	4.50	2.12	7.80	5.29	2.30	2.00	8.00	2.83
Radio/Music	7.63	9.13	3.02	6.78	11.69	3.42	5.00	32.00	5.66
Education	8.88	6.13	2.47	9.60	0.93	0.97	10.00	0.00	0.00

^1^Range: 0(*none*) to 10(*high*)

### Apparatus

The experiment was programmed with the Psychophysics toolbox (Brainard, 1997) in Matlab (version 2022b, The Mathworks Inc., Natick, MA, USA). A video-based Eyelink 1000 eye-tracker in desktop-mount mode (SR Research, Ontario, Canada) was used to record eye movements, with a sampling rate of 1,000 Hz. Stimuli were displayed on a screen with resolution of 1,920 × 1,080 pixels and projected with a VPixx PROPixx projector (VPixx Technologies, Saint-Bruno, Quebec, Canada) set to a refresh rate of 120 Hz.

### Word stimuli

In this study, a set of 800 five-character word stimuli were selected from the English Lexicon Project by Balota et al. (2007)**.** Our stimulus design adhered to the recommendation of Rayner (1979), ensuring that the word stimuli were at least four characters long (in our case, five characters). The average size of these word stimuli was measured to be 90.7 pixels (SD = 10.9) horizontally and 29.4 pixels vertically (SD = 4.2)*.* We exclusively included verbs and non-inflected nouns, focusing on these specific word categories. The selected word set encompassed high-, medium-, and low-frequency words, covering a broad range of occurrences, with an average hyperspace analogue to language (HAL) frequency of 15,263.885. HAL is a computational model that represents the semantic relationships between words based on their co-occurrence patterns in large text corpus. Therefore, it is a valid and reliable measure to assess the categorization of words based on their frequency (Burgess, 1998). Each word stimulus within these groups was deliberately chosen to exhibit orthographic neighbors, demonstrating an average of 3.7 such neighbors and an Orthographic Levenshtein Distance (OLD) measure of 1.8. The OLD is a metric used to assess the orthographic and spelling similarity between two strings of characters (Yarkoni et al., 2008). We divided the word list equally into two distinct groups: 400 target words and 400 probe words for the memory test. We also created an additional set of 120 practice words, comprising 60 targets and 60 probes. The practice word stimuli, which were used during the training/familiarization stage prior to the main experiment, were selected using the same criteria as the main experiment word stimuli. Word stimuli subtended approximately 1.52 ± 0.20 dva (degree of visual angle; M ± SD) as viewed by participants**.**

### English reading

To assess the English reading abilities of the participants, we employed a combination of self-report measures and objective assessments. Firstly, participants completed the Language Experience and Proficiency Questionnaire (LEAP-Q), which provided self-reported ratings of speaking, listening, and reading proficiency (Marian et al., 2007). The questionnaire was presented online using Qualtrics survey software (https://www.qualtrics.com) prior to the commencement of the experiment.

In addition to the self-report measures, we implemented an objective measure by assigning participants a short reading task, immediately following the LEAP-Q questionnaire. The passage was sourced from the official practice test of the Scholastic Aptitude Test (SAT) by the College Board (2021), itself taken and adapted from Michael Slezak, “Space Mining: the Next Gold Rush?” © 2013 By New Scientist, and consisted of 335 words and two comprehension questions. The SAT is a standardized test that assesses reading comprehension skills therefore it is a valid and reliable measurement of reading skills (Hannon & McNaughton-Cassill, 2011). The SAT reading passage was appended to the end of the LEAP-Q. The time to read the text was recorded for each participant and converted into a words-per-minute reading speed for each participant (see Table 2 below). All the participants read the passage within the expected reading time for proficient readers of English (Rayner et al., 2001).

**Table 2:** Descriptive statistics of reading rate (words per minute) across English dominance groups

	Reading Rate (words per minute)		
	Mean	Var	SD
Overall	371.14	22209.35	149.03
English 1st Dominant	405.39	7938.05	89.10
English 2nd Dominant	378.50	32130.80	179.25
English 3rd Dominant	197.33	6889.03	83.00

### Procedure and design

**Fig. 1:**
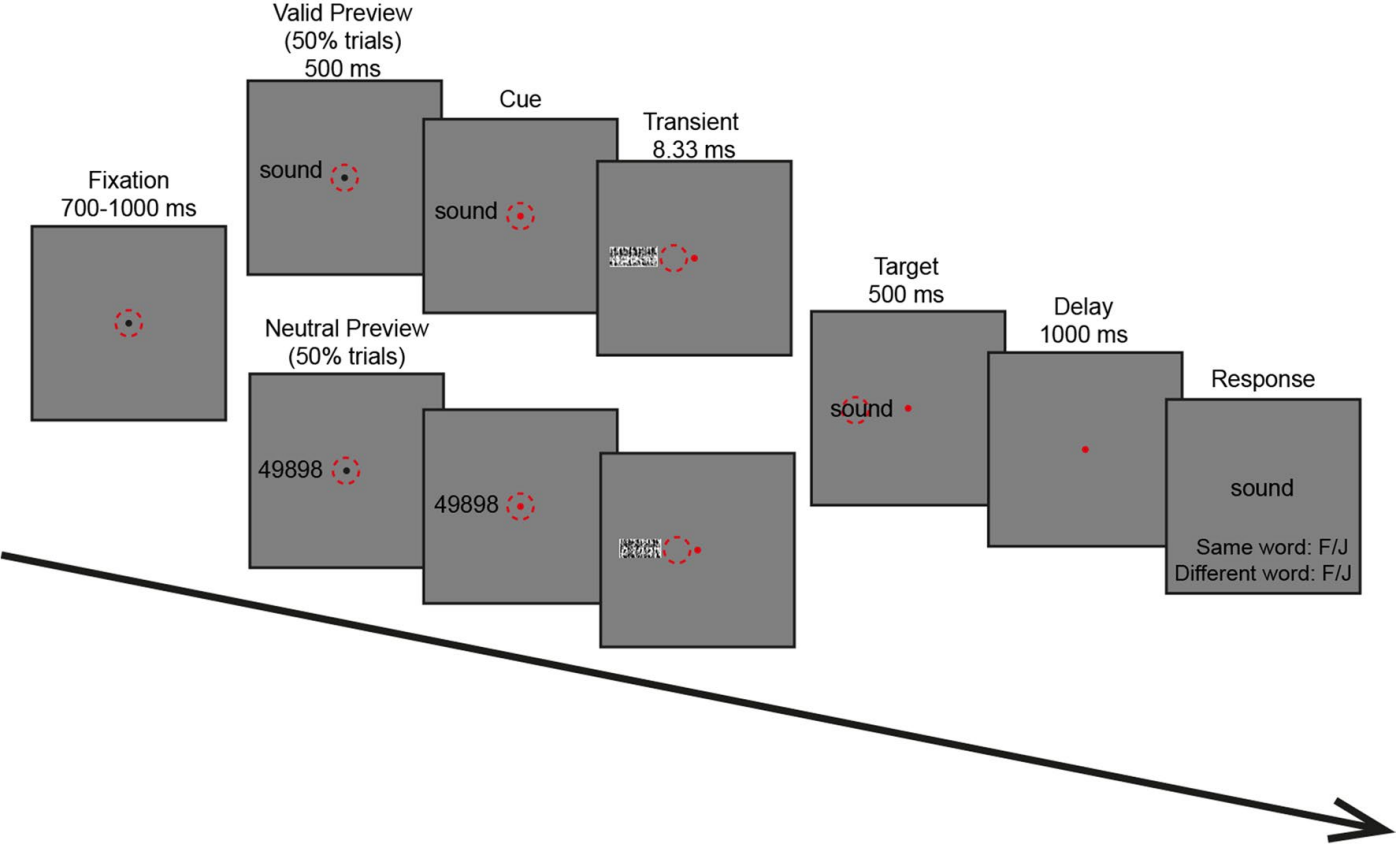
Illustration of the trial sequence. Participants maintained their gaze on a central fixation point and then a stimulus appeared in their parafoveal vision in either the right or left visual field. After a brief preview period, the fixation dot changed color, indicating that participants should make an eye movement towards the target. Then, after the saccade was detected, there was a brief transient for one frame and then the target word was presented for 500 ms. After target disappearance, there was a blank delay followed by the presentation of a word (memory probe) on the screen. The word was the same as the target on 50% of trials

Participants sat in a chair and placed their head on a chin rest at a distance of 90 cm from a projection screen. The experiment took place in a dark, magnetically shielded room. The time course of events during a trial, for both valid and the invalid conditions, is illustrated in Fig. 1. Each trial started with a central fixation dot (diameter = 0.25 dva). Participants maintained central fixation, which was monitored online by the eye-tracker. The trial restarted every time the participants deviated from the fixation dot by more than 1.5 dva. After 700–1,000 ms of stable fixation (fixation duration randomly selected on each trial), the parafoveal preview stimulus, which subtended approximately 1.5 dva, appeared on-screen in either the left or the right visual field (randomly interleaved) at a distance centered at 3 dva from central fixation. On 50% of trials (the invalid preview condition), the preview stimulus was a number string of similar length to the subsequently foveated target word. In the valid preview condition, the preview stimulus was identical as the target word. The participants were asked to stabilize their gaze on the fixation despite the onset of the stimulus in the periphery. After 500 ms of parafoveally previewing the stimulus, the fixation dot turned red, cueing the participants to make an immediate saccade to the parafoveal stimulus located in their right or left visual field.As soon as a valid saccade (i.e., when two consecutive gaze samples were at least 0.12 dva apart on the screen) was recorded, a transient mask was displayed for one frame (8.33 ms). The transient was the phase-scrambled version of the preview stimulus, served to mask any possible changes made to the preview stimulus during the saccade. Participants would not perceive the mask due to saccade suppression (for review, see Ross et al., 2001). The mask was followed by the target word, which remained for 500 ms. Then, there was a blank delay of 1,000 ms, followed by the memory probe word. In 50% of the trials, the memory probe matched the target. The participants were asked to indicate whether this was the same or different word by pressing the corresponding key (F/J) on a standard keyboard. Once this step was completed a new trial began.

Participants performed 400 trials divided into eight experimental blocks that contained 50 trials each. Valid and invalid previews presented on the left and right side of the screen were randomly intermixed between trials to control for order effects. The words used in each condition were randomized for each participant, so there was no systematic difference in word stimuli across conditions. At the start of the experiment, the eye-tracker was calibrated with a 13-point grid. Throughout the experiment, the eye tracker monitored the recorded gaze position online. If the calibration ceased to be correct, the experiment was put on hold and the eye tracker was recalibrated. Each participant ran at least two practice blocks (ten trials each block) with the same conditions as in the proper experiment. They proceeded in the proper experiment once they scored more than 90% accuracy in the memory task in at least two consecutive practice blocks.

### EEG and eye-tracking data recording and analysis

The EEG was recorded in an electromagnetically shielded booth, using a 32-channel DC system (Fp1, Fp2, F7, F3, Fz, F4, F8, FC5, FC1, FC2, FC6, T7, C3, Cz, C4, T8, TP9, CP5, CP1, CP2, CP6, TP10, P7, P3, Pz, P4, P8, PO9, O1, Oz, O2, PO10) (Brain Products GmbH, software: Brain Vision Recorder version 1.21) at a 1,000-Hz sampling rate. The placement of the electrodes followed the 10–20 system, the ground electrode was placed at Cz, and the online reference at Fz. All impedances were kept < 5 kOhms. A video-based Eyelink 1000 eye-tracker in desktop-mount mode (SR Research, Ontario, Canada) was used concurrently with the EEG to record eye movement at the sampling rate of 1,000 Hz. Parallel port triggers were sent to both EEG and eye-tracking acquisition systems simultaneously by means of a splitter cable. To synchronize both data streams offline, we used the EYE-EEG add-on (Dimigen et al., 2011) to the EEGLAB toolbox (version 14.1.1, Delorme & Makeig, 2004). We processed all data using Matlab (versions 2019a and 2023a, The Mathworks Inc.).

### EEG-EYE synchronization

Event markers from the EEG and eye-tracking data were synchronized offline using the EYE-EEG extension (Dimigen et al., 2011) in the EEGLAB toolbox (version 14.1.1, Delorme & Makeig, 2004). All event markers in both systems were aligned and the eye position and event data were imported into the EEG. To synchronize, the eye-tracking data between the start- and end-event was linearly interpolated to match the number of EEG sampling points recorded during the same time interval. Afterwards, shared events were identified and a linear function fitted to the shared event latencies in order to refine the estimation of the latency of the start-event and end-event in the eye-tracking recording. Saccades under 10 ms were excluded from the data. Finally, we tested the accuracy of the synchronization between the eye-tracking and EEG data using cross-correlation between the horizontal eye position and the EEG at frontal channel Fp2. This analysis showed 1 ms or less of delay between eye-tracking and EEG signals across participants.

### EEG preprocessing

After synchronizing the eye-tracking and EEG events, the data were down-sampled to a sampling rate of 250 Hz and band-pass filtered between 0.1 and 40 Hz. To identify channels in the data that might contain excess noise or artifacts, we first employed an automated function to initially detect bad channels prior to visual inspection. To achieve this, we first extracted data from the scalp channels (1:32) and reshaped it into a matrix, which was then z-scored across all channels and data points to normalize. The z-scored data were reshaped back into its original format, with the number of channels and time points and standard deviation computed for each channel. Next we compared the channels against an established z-score threshold of 2 standard deviations. Channels with a standard deviation above this threshold are considered potentially problematic or noisy (Delorme & Makeig, 2004). We then visually inspected the dataset to establish a list of bad channel indices and stored them. We then re-referenced the channels to the average reference. The bad channels were removed and interpolated using a spherical interpolation method (Huber-Huber et al., 2019).

To further minimize the potential impact of oculomotor artifacts from the EEG signal, we applied Independent Component Analysis (ICA) (Makeig et al., 1995).The ICA was conducted on non-baseline-corrected continuous data that had previously undergone manual inspection to locate, identify, and exclude major artifacts. We performed a separate pipeline ICA instead of using the main preprocessing in order to maintain temporal precision and avoid data distortion (Dimigen, 2020). We used a high-pass filter Hamming windowed sinc finite impulse response (FIR), setting the passband edge at 2 Hz. This filtering step was applied after down-sampling and before low-pass filtering, following the methods of Dimigen et al. (2011) (cf., Winkler et al., 2011).

Following the Independent Components Analysis, the continuous EEG data was epoched into trials (from -200 to 600 ms) and binned according to the experimental condition. Each bin contained the four experimental conditions (left-valid, right-valid, left-invalid, right-valid). Artifacts in the EEG data were detected and rejected using parameters set to flag artifacts: applying a low-pass filter with a cut-off frequency of 30 Hz, and rejecting data points outside the threshold range of -100 to 100 microvolts. Artifact rejection was performed within the time window of -200 to 600 ms. Following this, the trials were further visually inspected for additional artifacts and confirmed the automated artifact findings. Trials that were marked as bad according to the artifact rejection criteria were excluded from further analysis.

In line with previous studies on fixation related potentials in reading research (Huber-Huber et al., 2019; Kornrumpf et al., 2016), we performed a baseline correction with a 200-ms pre-fixation (ERP) and target period (FRP). This was done to correct any differences in signal offset due to the different preview conditions. After artifact rejection and trial exclusion, the individual ERPs were computed using ERPLAB functions (Lopez-Calderon & Luck, 2014). The preprocessing of data was concluded with grand average ERPs for Preview and Target conditions.

### Statistical analysis

Statistical comparison between the preview conditions was achieved with a bootstrap t-test between the valid and invalid FRPs across all channels and time-points. In order to test for an interaction effect, the waveform differences between left and right conditions were similarly compared for valid and invalid trials. The same analysis was applied to test for significant differences between ERPs locked to words and number preview stimuli. Statistical significance was established with non-parametric methods and all comparisons were corrected for multiple comparisons with cluster-based methods as implemented in the Fieldtrip toolbox (Maris & Oostenveld, 2007). EEG preprocessing was implemented with EEGLAB, ERPLAB, and the EYE-EEG toolbox. Statistical testing was implemented with Matlab custom scripts and the Fieldtrip toolbox (Oostenveld et al., 2011).

Multivariate pattern analysis (MVPA) was implemented using ADAM toolbox (Amsterdam Decoding and Modelling Toolbox; Fahrenfort et al., 2018) on MATLAB 2019a (Mathworks Inc.), with the aim to decode neural patterns elicited by the preview stimulus (i.e., word vs. number) and, more importantly, by the post-saccadic condition (i.e., valid vs. invalid trials). In detail, Standard Linear Discriminant Analysis (LDA) was performed for all comparisons at each time point, using a tenfold cross-validation procedure, considering data from all the electrodes. In this procedure, data for each comparison were split into ten equal folds, and classification was performed by training on nine and testing on the remaining fold. Each individual fold was used once for testing, and then the performance was averaged across each of these tests. Due to the different number of trials in the different experimental conditions, we performed oversampling using the ADASYN method (Fahrenfort et al., 2018). ADASYN corrects for class imbalances by generating synthetic data for the experimental condition with fewer trials, using a weighted algorithm guaranteeing that synthetic trials are close to the decision boundary (for a detailed description of these methods, see Fahrenfort et al., 2018; He et al., 2008). Classification performance was measured using the area under the curve (AUC; e.g., Fahrenfort et al., 2018).

In a first step (i.e., diagonal decoding), we trained and tested the classifier on the same time points, probing decoding performance against chance (50%) to judge the time points at which the classifier was successfully able to differentiate between experimental conditions, using right-sided cluster-based permutation t-tests with 2,500 iterations (above chance = 0.5; Maris & Oostenveld, 2007). To investigate spatial differences in the decoding of neural activity elicited by different experimental conditions, we computed topographical scalp maps based on classifier weights for individual features (i.e., electrodes). In detail, we multiplied the classifier weights with the data covariance matrix, thus evaluating the neural pattern as neural sources resulting from the comparison between different experimental conditions (for a detailed description of this method, see Haufe et al., 2014). The activation pattern of each subject was spatially normalized by subtracting the mean across electrodes and dividing by the standard deviation. The resulting topographic maps represented z-scores with average 0 and unit standard deviation, showing the spatial distribution of the neural activity that underlies successful discrimination between experimental conditions (Fahrenfort et al., 2017).

### Temporal generalization

In a second step, we performed temporal generalization analysis using cross-classification across time (i.e., off-diagonal decoding; King & Dehaene, 2014), with the aim of disentangling whether the neural code associated with specific activation patterns identified at given time points was recurrent and generalized at subsequent timepoints, clarifying whether multiple neural stages were involved during preview and post-saccadic stimuli processing and how their neural codes were unfolded over time. Accordingly, temporal generalization allowed us to unveil whether neural dynamics are persistent or transient over time, offering a way to identify the time course and the longevity of the neural representation of processed stimuli (Cichy & Oliva, 2020; King & Dehaene, 2014). This is represented using a color-map where the y-axis represents training times, and the y-axis represents testing times. In this graphical representation, the diagonal axis shows the training and testing classifier performance on the same time points (i.e., diagonal decoding) and the resulting off-diagonal points shows how well the pattern of neural activity is generalized to other time points (i.e., temporal generalization). The classifier was trained and tested by computing the same procedures as the diagonal decoding analysis presented above, but in this case above-chance classification was measured implementing repeated testing for each time point and 2,500 iterations of cluster-based permutation testing to correct for false positives.

### Correlational analysis

Finally, we performed a Pearson correlation to investigate potential relationships between the decoded patterns of neural activity elicited by the preview stimulus (i.e., word vs. number) and by the post-saccadic condition (i.e., valid vs. invalid trials), with the aim of disentangling whether the processing of the preview stimulus may potentially impact the neural processing of post-saccadic stimulus. Accordingly, similarly to some recent studies (Farran et al., 2020; Mares et al., 2020; Marsicano et al., 2023), exploiting the greater analysis sensitivity of MVPA technique in unveiling individual pattern of neural activations (Grootswagers et al., 2017; Marti & Dehaene, 2017), we indexed from diagonal decoding analyses the following different key decoding parameters allowing a deeper investigation of the neural activation profiles associated with the decoding of the experimental conditions at the individual level: (i) decoding onset latency, i.e., the first time point at which decoding becomes significant; (ii) decoding peak latency, i.e., the time point at which accuracy (AUC) is highest; (iii) decoding peak accuracy, i.e., the highest decoding accuracy value (AUC); and (iv) decoding sustainability, i.e., measured as the mean of accuracy (AUC) on significant timepoints decoded over time. Importantly, these neural decoding measures were also correlated to oculomotor (i.e., mean amplitude and RTs of the saccades to the target and reading measures (words per minute reading speed in the separate reading task), probing whether decoding activity may predict behavioral performance.

## Results

### Univariate analyses

We first analyzed the evoked response to the onset of the preview stimulus while the participant maintained their gaze at the central fixation. This analysis served to check that the participant was processing the preview stimulus and to see how the ERP differed between the two preview types (word vs. number string). Also, given that the stimulus was presented outside the fovea, we were interested in the timing of the evoked responses given that most EEG studies of words have presented the stimulus at the fovea.

**Fig. 2:**
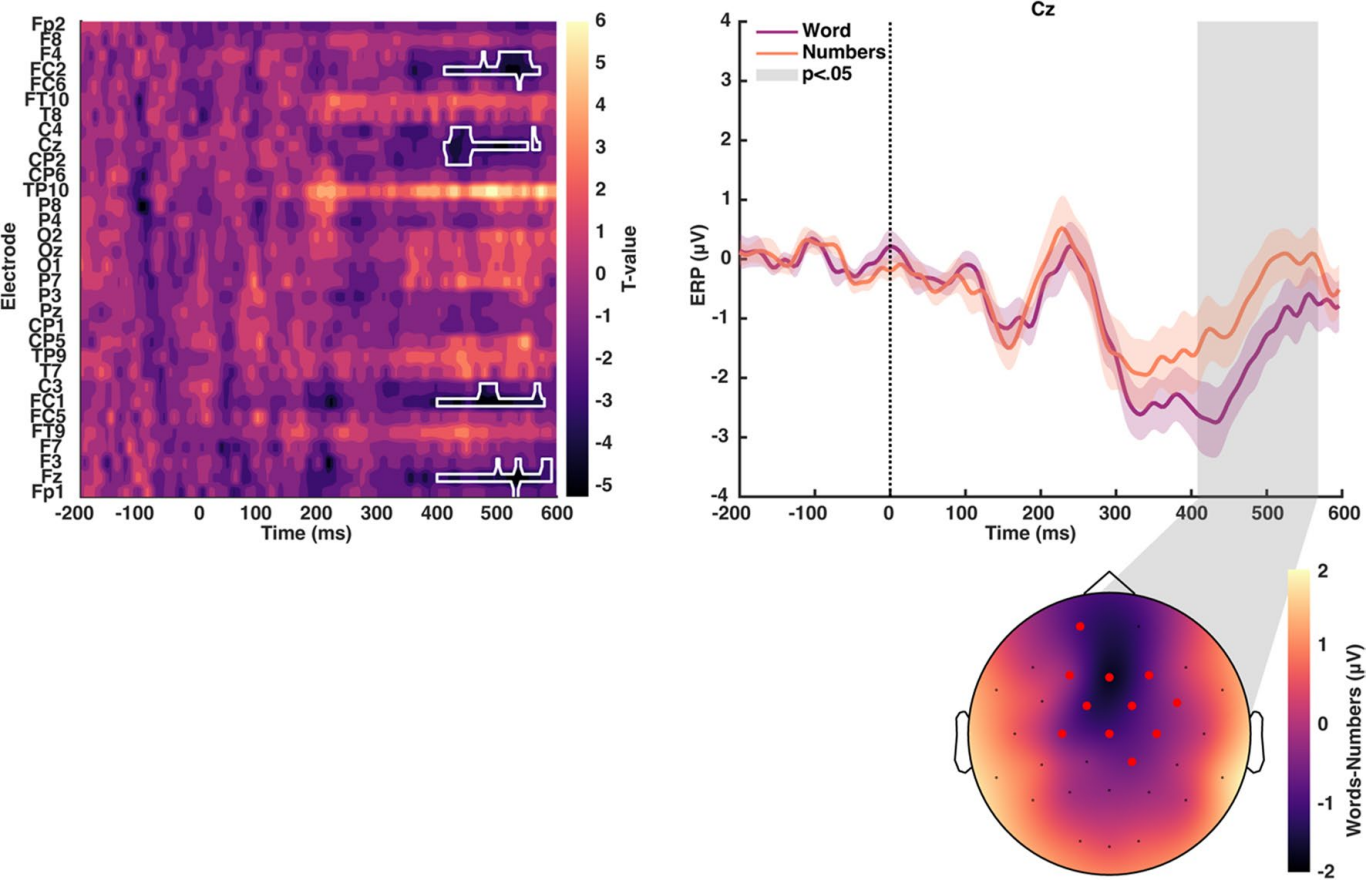
Event-related potentials (ERPs) of words vs. number preview stimuli. Comparison between neural activity elicited by written words and numbers as preview stimuli. (**Left**) T-values map for the comparison between conditions across all channels and time-points. The white outline indicates statistically significant differences (*p* < .05). (**Right**) Grand average ERP at channel Cz. Shaded areas reflect the standard error of the mean. Significant differences are outlined in grey and connected to their topographic maps in the bottom. Red markers indicate significant channels. Please note that negative mV values are presented below and positive numbers above the zero, in line with conventions in visual perception studies, rather than with “negative up - positive down” as in many studies of word processing

**Fig. 3:**
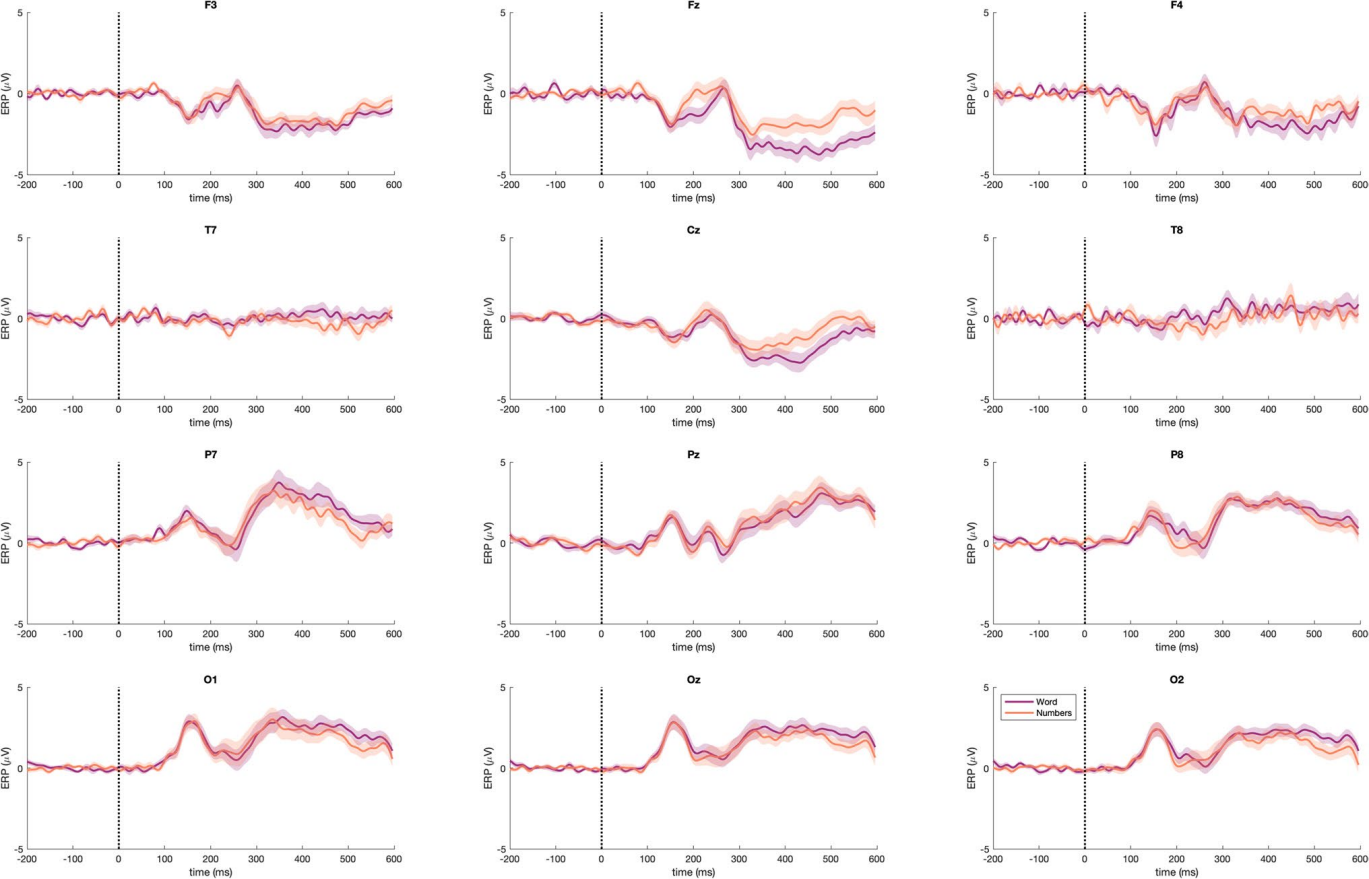
Event-related potentials (ERPs) for words vs. numbers across the scalp. ERPs of word and number stimuli at frontal (first row), central/temporal (second row), parietal (third row), and occipital (fourth row) channels

**Fig. 4:**
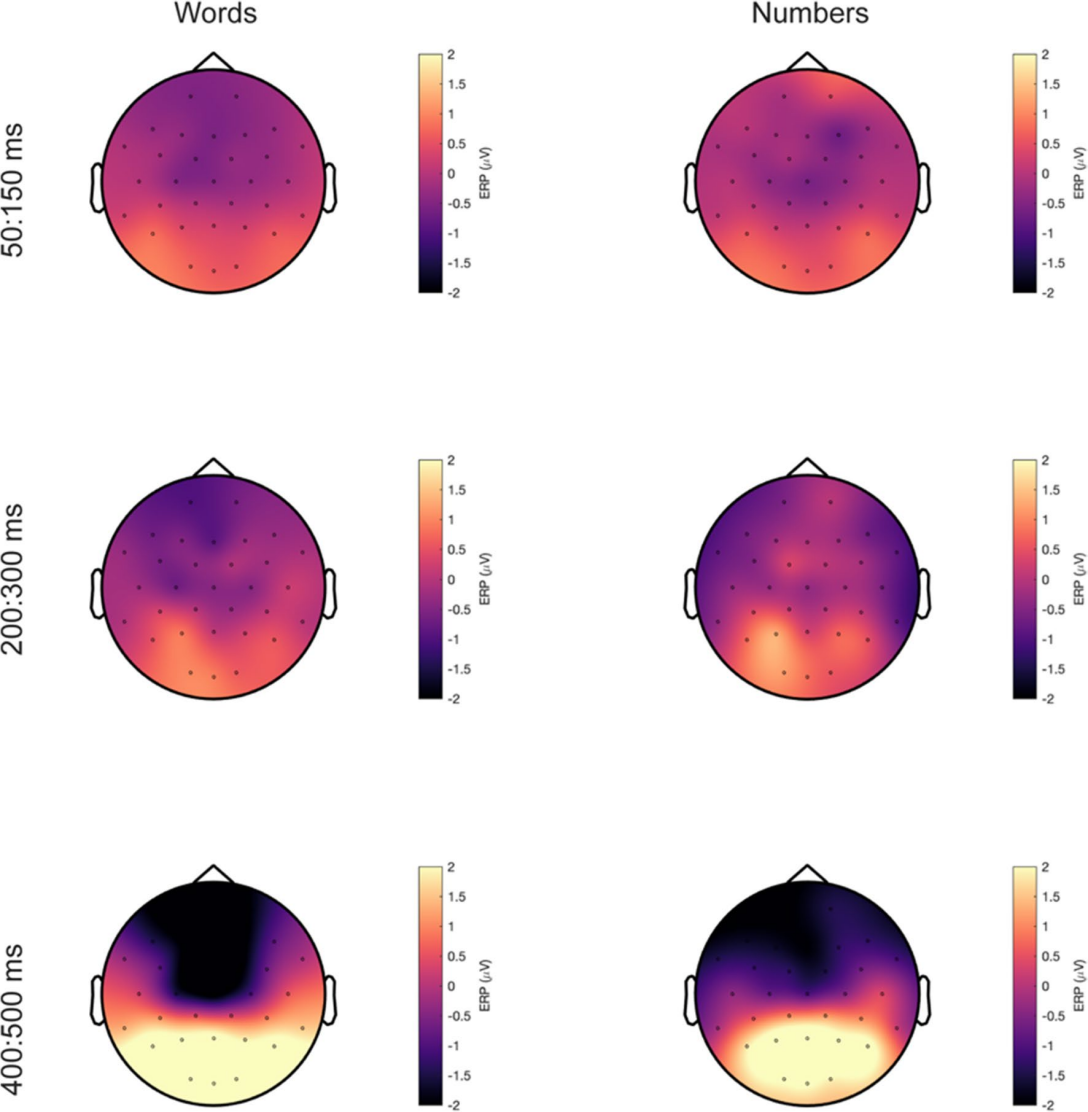
Topographic maps for words and numbers event-related potentials (ERPs). ERP responses for words (**left**) and number (**right**) averaged over 50–150 ms (top), 200–300 ms (middle), and 400–500 ms (bottom)

As expected, the onset of the preview stimulus generated a strong evoked response (Fig. 2). The initial response to words and numbers was quite similar in terms of the ERP. Only after around 300 ms was there a significant difference in the evoked response between the two preview stimulus types. The differences were mainly found in the more anterior electrodes (Figs. 3 and 4). This confirms that, at a basic visual processing level, the initial response to the two preview stimuli was similar. The differences, starting at 300 ms and strongest in the 400- to 500-ms time window, are consistent with participants extracting the category difference (word versus number strings) from the parafovea. We found no interaction effects with the stimulus side, left or right of center (all *p* > .05).

**Fig. 5:**
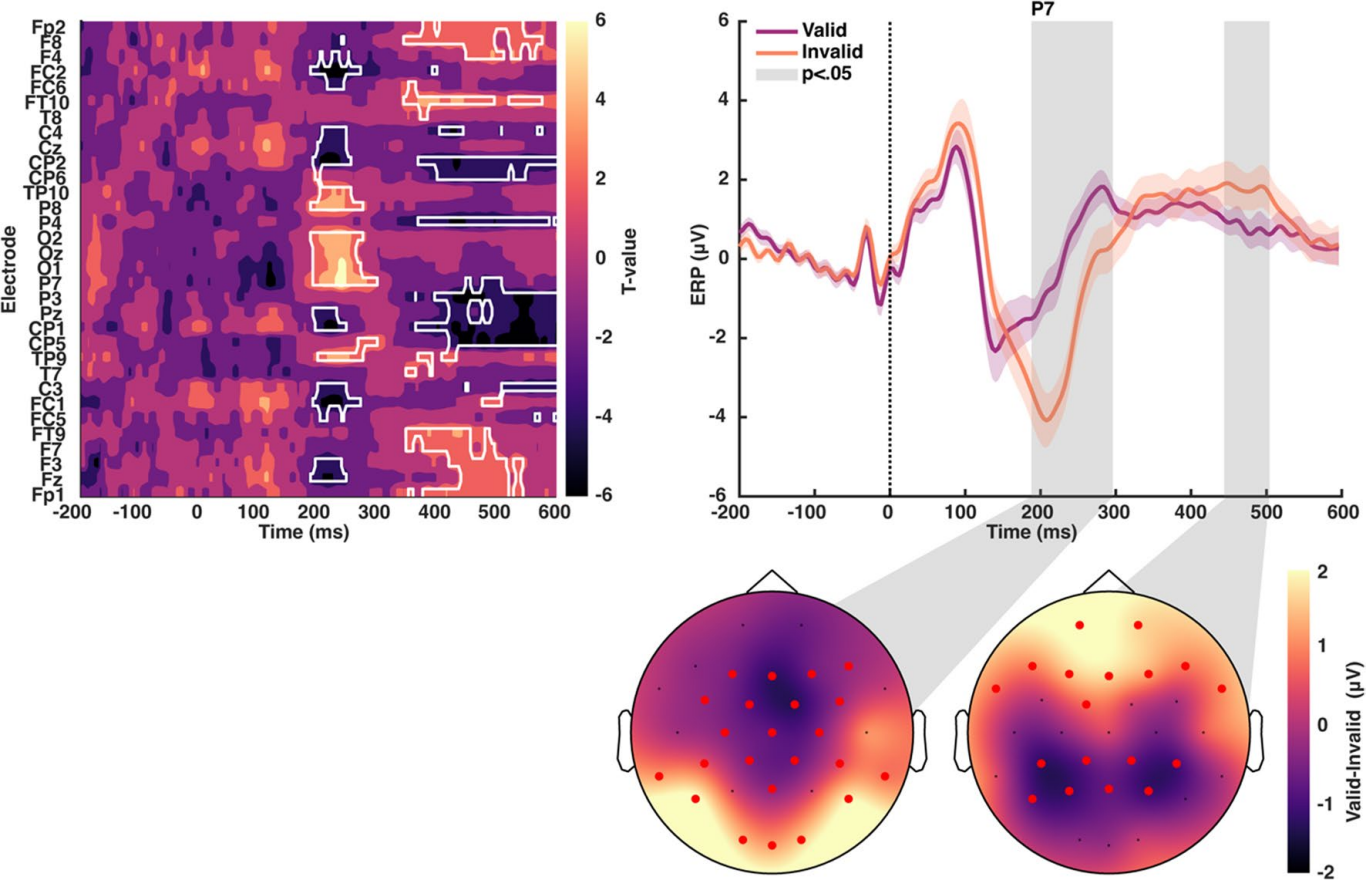
Fixation-related potentials (FRPs) for valid and invalid conditions. FRPs: comparison between neural activity of the valid and invalid conditions. (**left**) T-values map for the comparison between conditions across all channels and time-points. The white outline indicates statistically significant differences (*p *<. 05). (**right**) Grand average fixation event-related potentials (fERPs) at channel P7. Shaded areas reflect the standard error of the mean. Early (~200 ms) and late (~400 ms) significant differences are outlined in grey and connected to their topographic maps in the bottom. Red markers indicate significant channels. Please note that negative mV values are presented below and positive numbers above the zero, in line with conventions in visual perception studies, rather than with “negative up - positive down” as in many studies of word processing

**Fig. 6:**
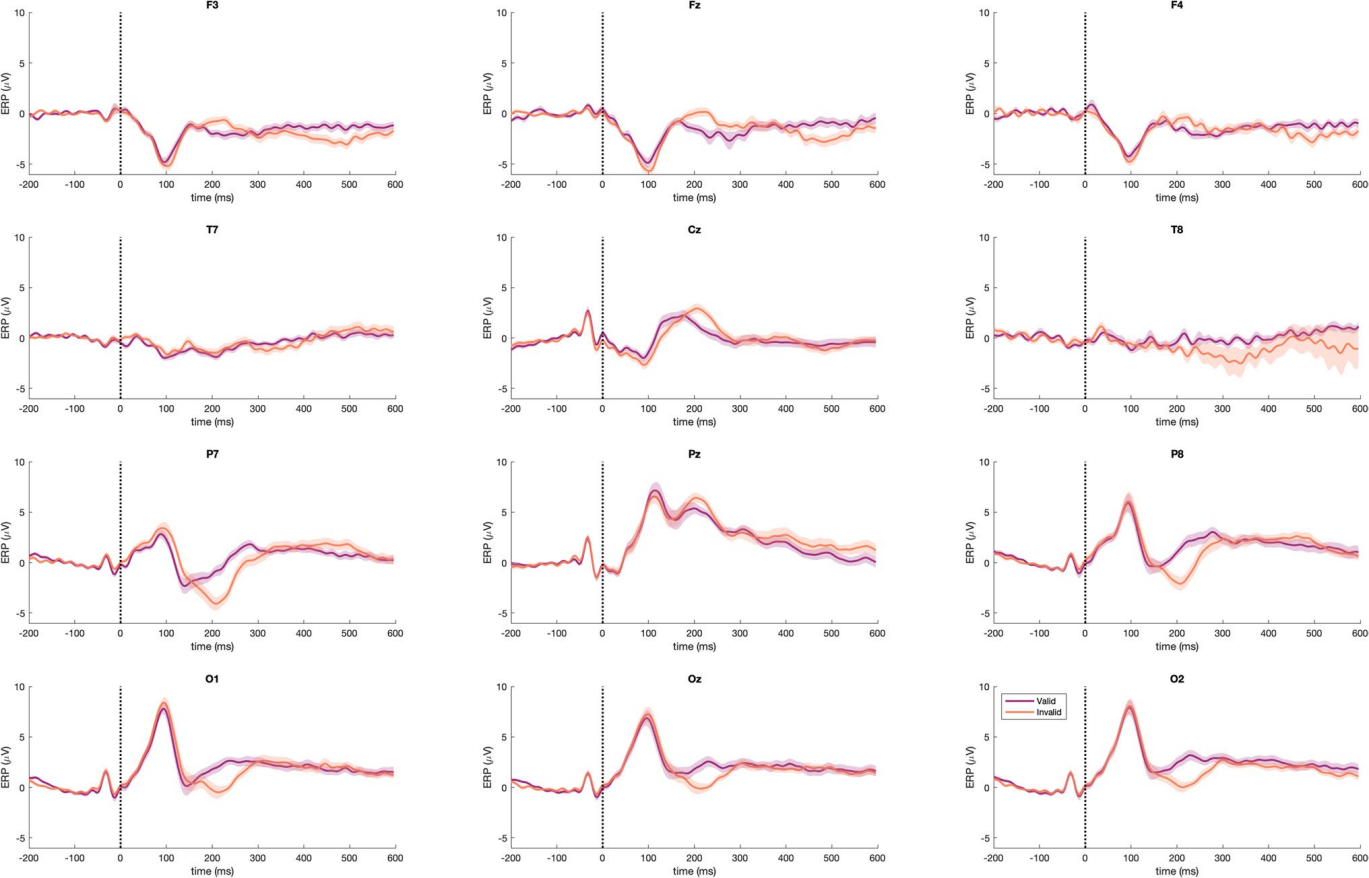
Fixation event-related potentials (fERPs) for valid vs. invalid trials across the scalp. fERPs of valid and invalid trials at frontal (first row), central/temporal (second row), parietal (third row), and occipital (fourth row) channels

**Fig. 7:**
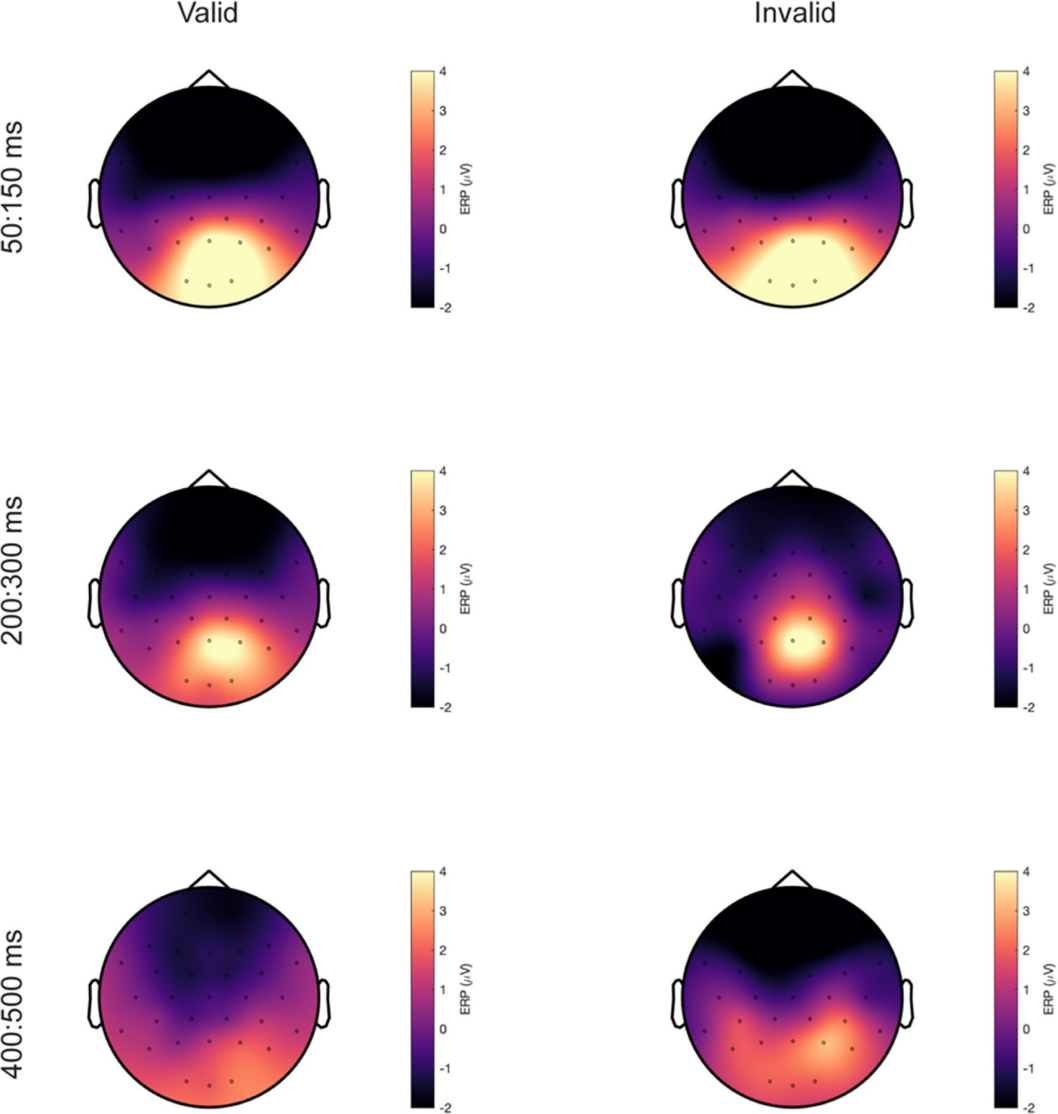
Topographic maps for valid and invalid fixations event-related potentials (fERPs). fERP responses for valid (**left**) and invalid (**right**) trials averaged over 50–150 ms (top), 200–300 ms (middle), and 400–500 ms (bottom)

Next, we calculated the FRP to the word stimulus to determine how it was influenced by the preview stimulus type (valid/word or invalid/number string). As shown in Fig. 5, there was a strong FRP to the word target. When there was an invalid/number string preview, there was the expected, typical evoked response to a word stimulus as found in previous studies of sentence reading (Dimigen et al., 2012; Kornrumpf et al., 2016). This response was particularly evident in the time period around 200–300 ms post-saccade. In contrast, with a valid/word preview, the FRP was dramatically reduced in the 200- to 300-ms window. Specifically, the initial positive deflection (as illustrated in electrode P7 in Fig. 3) was similar for both the valid and invalid preview conditions. However, in the 200- to 300-ms time window valid trials showed a greatly reduced evoked response. This difference between conditions was confirmed in a whole-scalp permutation test analysis (Fig. 5), showing clusters in frontal, central, parietal and occipital regions (see the bottom part of Fig. 5 for a topographical map of differences in the 200- to 300-ms time period). In addition, there was a second significant difference between conditions in the 400- to 500-ms time period, in particular in frontal and parietal electrodes, which is visible in the permutation tests (Figs. 5, 6 and 7) and shown in the topographic plot for that time period (Fig. 5, bottom right). The overall shape and magnitude of the preview effect was similar to that reported by Kornrumpf and colleagues (Kornrumpf et al., 2016) when comparing FRPs in parietal-occipital electrodes for valid preview versus no preview (those authors used the letter string “Xxxxx”).

As expected, participants followed the eye-movement instructions (on all of the trials included in the analysis). In terms of the saccades to the word targets, participants saccaded directly to the word, with a tendency to look near the word center (mean amplitude was 3.5° of visual angle). Mean saccadic reaction times were 289 ms (SD = 50.2 ms), showing that participants responded quickly but not as rapidly as might be expected if the cue and the target were in the same location. Participants were instructed to maintain their fixation on the target upon saccadic offset, in order to reduce the effects of subsequent saccades on the EEG signal. First fixation durations were around 367 ms (SD = 98.9 ms), consistent with participants following that instruction, maintaining fixation in preparation for the memory test.

### Multivariate analyses

#### MVPA of preview stimulus (words vs. numbers)

**Fig. 8:**
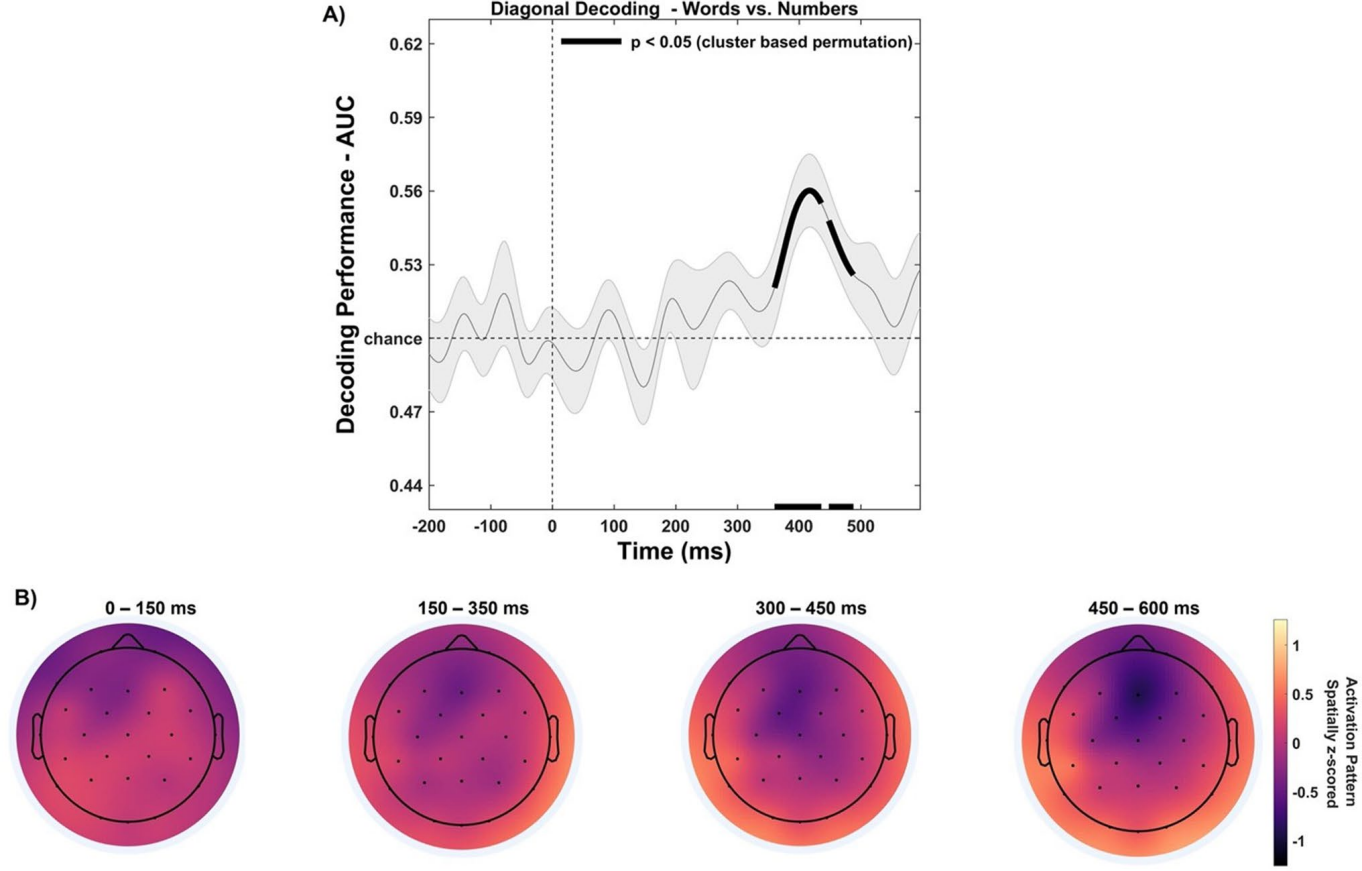
Preview-locked trials diagonal decoding results (word vs. number). (**A**) The diagonal decoding performed on all the electrodes of the preview stimulus neural response revealed that decoding accuracy (AUC) was sustained significantly above chance level in the 360- to 488-ms time window (solid lines; 50%). Vertical lines indicate preview stimulus onset (0 ms). The horizontal line indicates the chance level (50%) against which the performance was tested to judge at which timepoints the classifier was successfully able to differentiate between experimental conditions (right-sided cluster-based permutation t-tests; *p *< 0.05). Shaded areas around bars represent the standard error across participants. (**B**) Activation patterns derived from the product of the weight vectors and the covariance matrix for individual features (electrodes) over preview-locked time window. The resulting topographic maps represent z-scores, showing the spatial distribution of the neural activity that underlies successful discrimination between experimental conditions

First, we trained and tested the classifier to decode the neural pattern elicited by the preview stimulus (words vs. numbers) considering all electrodes on the scalp. Regarding diagonal decoding, the cluster-based permutation revealed that classification accuracy (AUC) was significantly above chance level (50%; Fig. 8, panel A), showing significant decoding of word vs. numbers beginning at around 360 ms and ending around 436 ms (*p *< 0.001), and subsequently in the 448- to 488-ms time window (*p *= 0.021), with the highest decoding accuracy (AUC) reaching 66.1 ± 4.8%, emerging at a latency of 405 ± 90.24 ms. This result is consistent with the previously reported univariate ERP analysis, both in terms of the time windows and the topographic distribution of the neural activity that underlies successful discrimination between word vs. number experimental comparison (Fig. 8, panel B), although the estimate of the time period of this difference emerged earlier in MVPA analysis with respect to univariate ERPs analysis (~50 ms). However, the temporal generalization analysis (i.e., cross-classification across time; King & Dehaene, 2014) did not reveal that training the classifier at a given time point effectively generalized to other time points during the preview-locked time window. This suggests that decoding was picking up on a transient, evoked response rather than persistent neural patterns.

### MVPA of post-saccadic stimulus (valid vs. invalid)

**Fig. 9:**
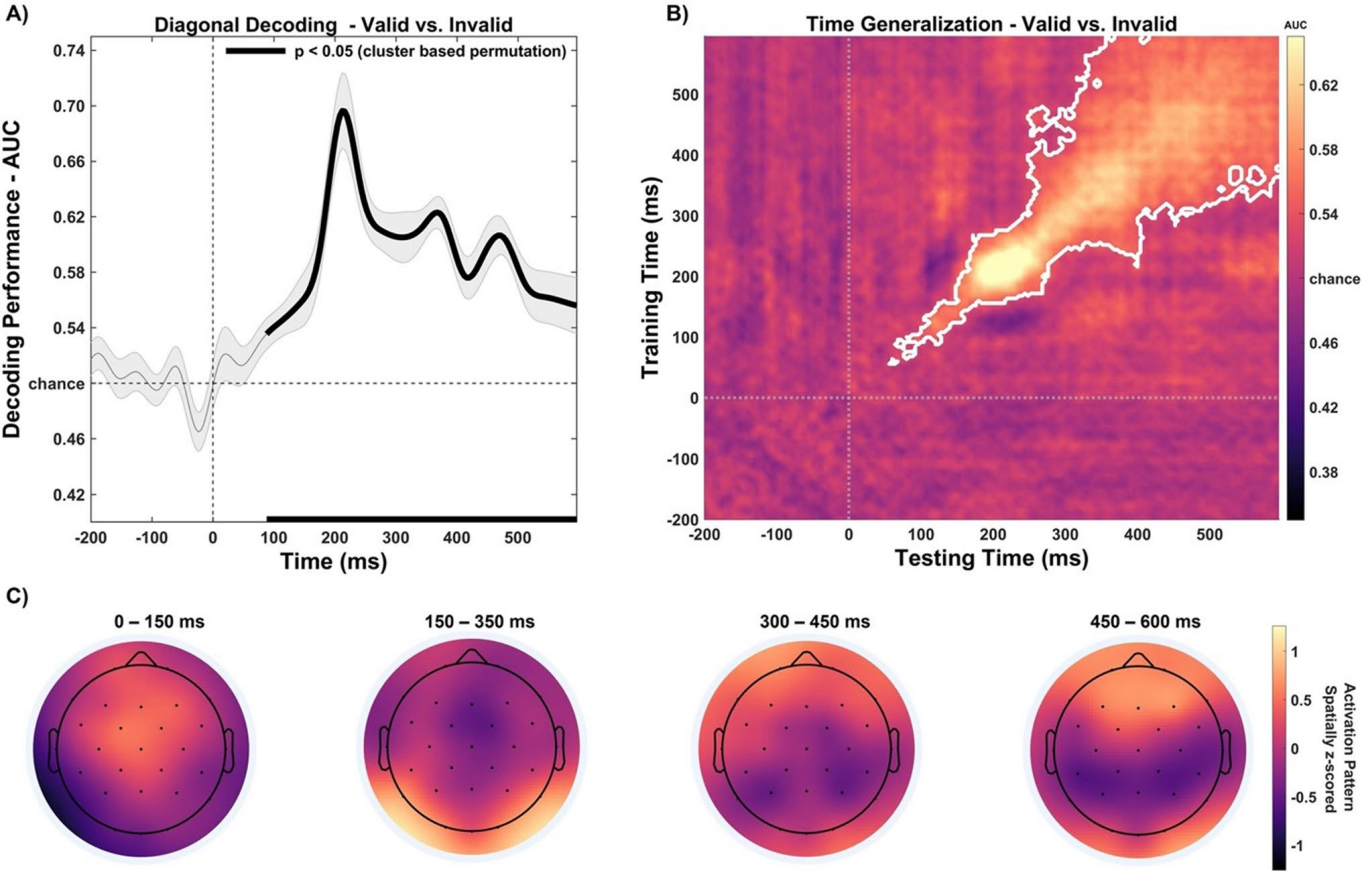
Post-saccadic stimulus diagonal decoding results (valid vs. invalid condition). (**A**) The diagonal decoding performed on all the electrodes of the post-saccadic stimulus neural response revealed that decoding accuracy (AUC) was sustained significantly above chance level from 88 to 596 ms (solid lines; 50%). (**B**) Time generalization analysis trained and tested on the post-stimulus time window. Results showed that training the classifier at a given time point generalized across a range of timepoints (e.g., statistically significant timepoints are outlined in dark red), revealing a generalized pattern of neural activity corresponding to a cluster beginning at 56 ms and ending at the post-saccadic time window offset at 596 ms, thus indicating a persistent and stable neural representation of post-saccadic stimulus information throughout the time window analyzed. (**C**) Activation patterns derived from the product of the weight vectors and the covariance matrix for individual features (electrodes) over preview-locked time window. The resulting topographic maps represented z-scores, showing the spatial distribution of the neural activity that underlies successful discrimination between experimental conditions. Vertical lines indicate preview stimulus onset (0 ms). The horizontal line indicates the chance level (50%) against which the performance was tested to judge at which timepoints the classifier was successfully able to differentiate between experimental conditions (right-sided cluster-based permutation t-tests; *p* < 0.05). Shaded areas around bars represent the standard error across participants

Importantly, our main interest in implementing MVPA analysis concerned the decoding of the neural activity elicited by the post-saccadic stimulus based on the nature of the preview stimulus presented (valid vs. invalid conditions; Fig. 9). Accordingly, when we trained and tested the classifier on the same time point (i.e., diagonal decoding), the cluster-based permutation revealed that classification accuracy (AUC) revealed a significant decoding of experimental conditions (Fig. 9), corresponding to a cluster in the observed data ranging over an extended period of time from around 88 ms to 596 ms (*p *< 0.001), with the highest decoding accuracy (AUC) reaching 75.2 ± 6.41%, emerging at a latency of 222 ± 61.81 ms. Hence, diagonal decoding analysis revealed that the timing of the neural processing related to post-saccadic stimulus was dramatically different from the univariate ERP analysis (i.e., early (~200 ms) and late (~400 ms) significant differences), demonstrating an early decoding of post-saccadic stimulus (~100 ms before), which was continuously processed throughout the time window, as reflected by a significant decoding classification (Fig. 9, panel A). This pattern of results is consistent with information about the preview (and thus the post-saccadic stimulus) being represented in neural activity earlier than would be expected from the univariate analyses described above. However, despite such relevant timing differences between MVPA and univariate ERP analyses, the waveform peaks and topographical distribution of the neural activity that underlies successful discrimination in the diagonal decoding analysis (Fig. 9, panels A and C) seems to overlap with the ERPs components identified in the univariate analysis between valid versus invalid conditions. This difference is found in an early visual evoked response to post-saccadic stimulus in central-posterior scalp areas, and in a late component elicited in a more central-anterior scalp region.

Importantly, to unveil the specific neural dynamics emerging from the MVPA analysis, we computed cross-classification across time (i.e., temporal generalization) to investigate the transient and persistent neural patterns elicited by the post-saccadic stimulus onset (Fig. 9, panel B), clarifying whether multiple stages are involved in post-saccadic stimulus processing and how these neural dynamics unfolded over time. That analysis revealed a significant, persistent and generalized pattern of neural activity corresponding to a cluster in the observed data beginning at 56 ms and ending at the post-saccadic time window offset at 596 ms (*p *< 0.001; Fig. 9, panel B), with a peak of decoding activity identified at 222 ms (i.e., reflecting diagonal decoding peak AUC), suggesting a stable neural representation of post-saccadic stimulus information. Such a significant above-chance decoding activity across-time, depicting a square pattern of temporal generalization, may indicate an early neural code associated to stimulus processing (~100–200 ms), which generalizes its activity over time reaching a stable neural representation (~300–596 ms).

### Correlational analyses results

We considered potential relationships between the decoded patterns of neural activity elicited by the preview stimulus (i.e., word vs. number), and behavioral performance. Specifically, we correlated individual decoding for the post-saccadic condition (i.e., valid vs. invalid trials), oculomotor and behavioral measures. In particular, key decoding parameters (see Method section) were correlated to individual differences in reading behavior (i.e., mean of reading time) and oculomotor performance (mean amplitude and reaction times of the saccade to the target), allowing us to investigate the neurocognitive profile associated with processing of preview and post-saccadic stimuli at the individual level.

**Fig. 10:**
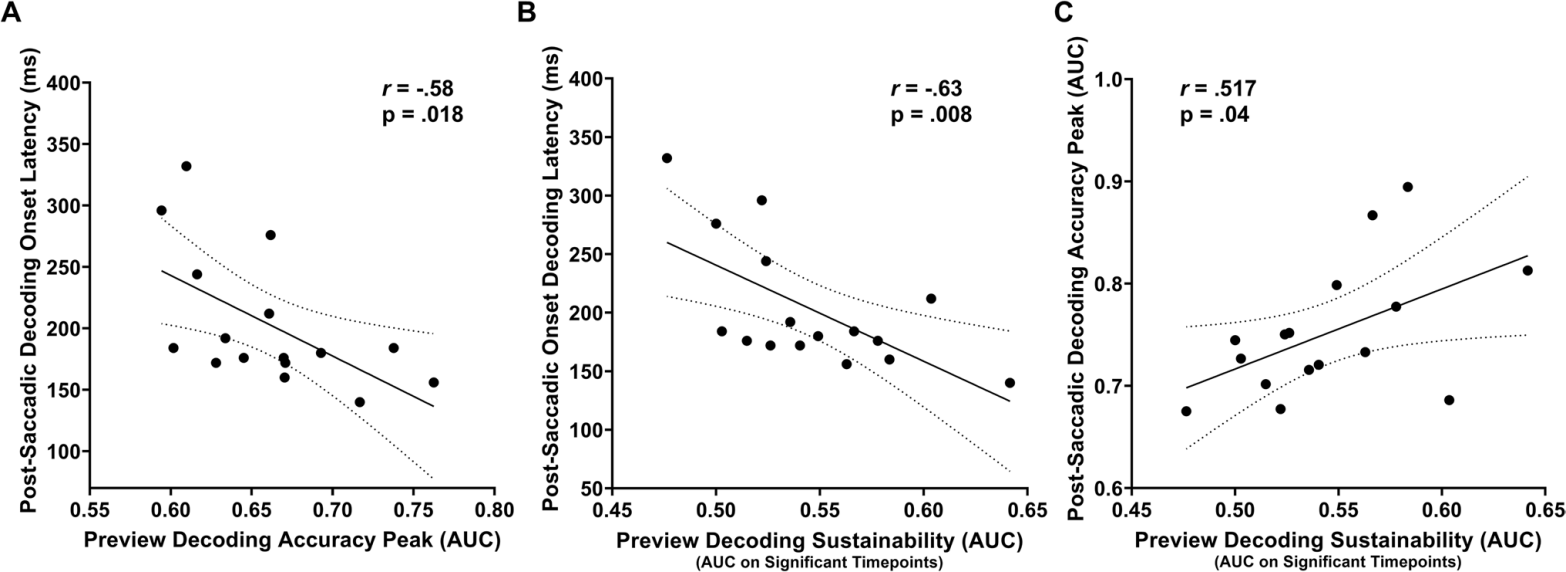
Significant Pearson correlations between preview and post-saccadic decoding measures. (**A**) Negative association between decoding accuracy (AUC) peak of the preview (word vs. number) and the decoding latency onset of post-saccadic condition (valid vs. invalid). (**B**) Negative relationship between the average decoding accuracy over the sustained significant period of preview stimulus (i.e., decoding sustainability) and the latency onset of decoding of post-saccadic condition. (**C**) Positive association between the average accuracy over the sustained decoding period of preview stimulus and post-saccadic decoding accuracy peak. Black dots represent individual datapoint; shaded gray lines represent 95% confidence intervals

First, we tested the hypothesis that a strong decoding of the preview stimulus would be related to the strength of the preview validity effect at the neural level (Fig. 10). There were three findings that provide support for this idea when considering single participant data. In terms of preview decoding peak accuracy, participants for whom the preview decoding was strongest (around 70–75%, for example) showed faster/earlier decoding onset latency of the preview validity for the post-saccadic stimulus (i.e., earlier processing of post-saccadic stimulus information), with a significant correlation (r = -0.580, *p *= 0.018). When looking at the average accuracy over the sustained decoding period, participants with strong and sustained decoding of the preview again showed an earlier post-saccadic onset latency (r = -0.63, *p *= 0.008) and a higher decoding accuracy (r = 0.517, *p *= 0.04). Thus, there was evidence that participants who more strongly encoded the preview showed a greater effect of that preview on post-saccadic stimulus processing.

**Fig. 11:**
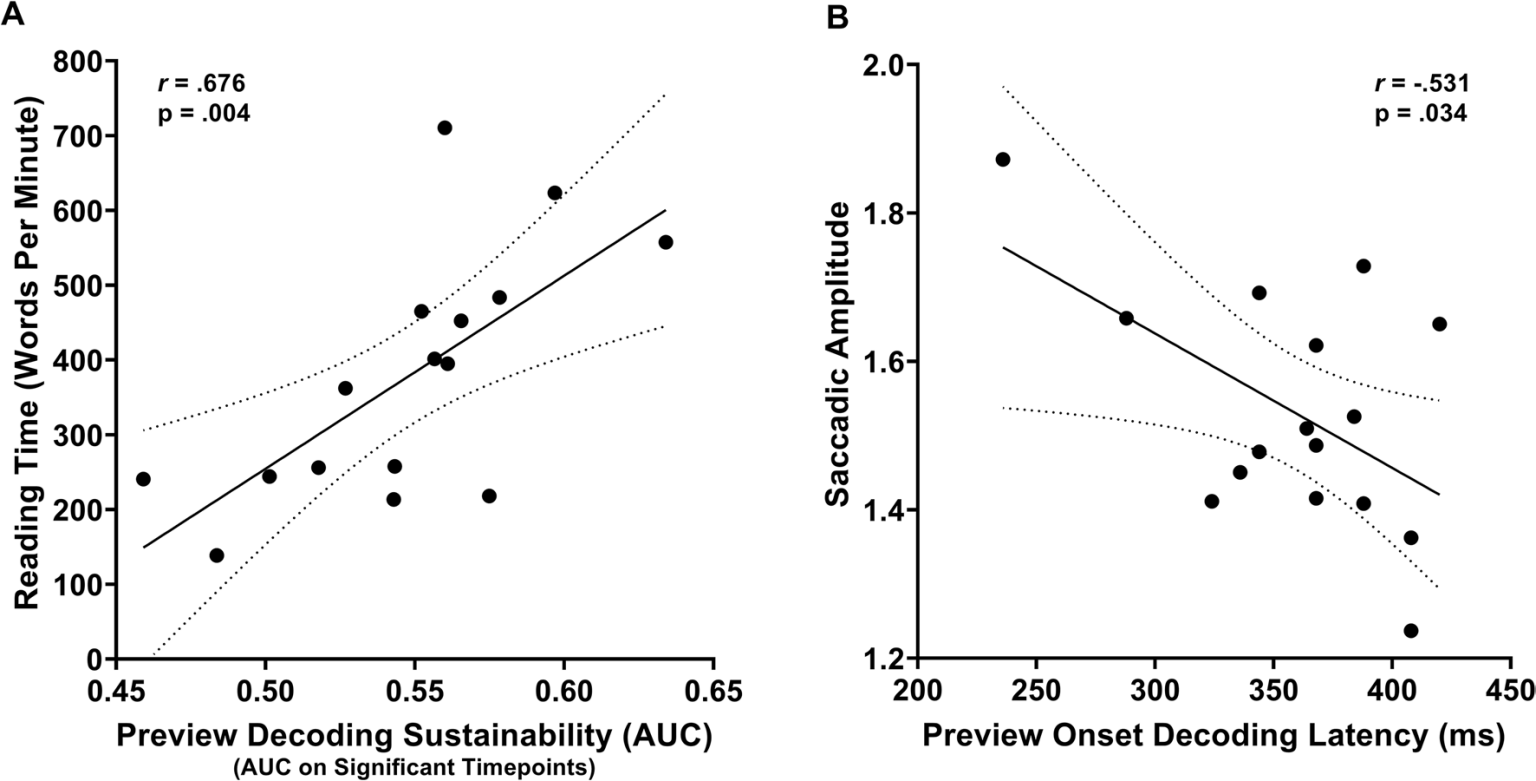
Significant Pearson correlations between preview and behavioral measures. (**A**) Positive association between the average accuracy over the sustained decoding period of preview stimulus (i.e., decoding sustainability) and reading time (i.e., words per minute). (**B**) Negative association between decoding latency onset of preview and the amplitude of saccades. Black dots represent individual datapoint; shaded gray lines represent 95% confidence intervals

Next, we considered the idea that, for individuals, the strength of their preview effect might be related to oculomotor (i.e., mean of the saccadic amplitude and latency to the target) and reading time (Fig. 11). In line with this prediction, participants showing higher average accuracies over the sustained decoding period presented faster reading times in terms of number of words read per minute (r = -0.616; *p *= 0.011). Furthermore, a later post-saccadic onset latency was negatively linked to the mean saccadic amplitude (r = -0.531, *p *= 0.034). To summarize, these results suggest that a faster and more pronounced decoding of preview stimulus may predict a functional processing of valid versus invalid post-saccadic stimulus conditions at the individual behavioral level.

**Fig. 12:**
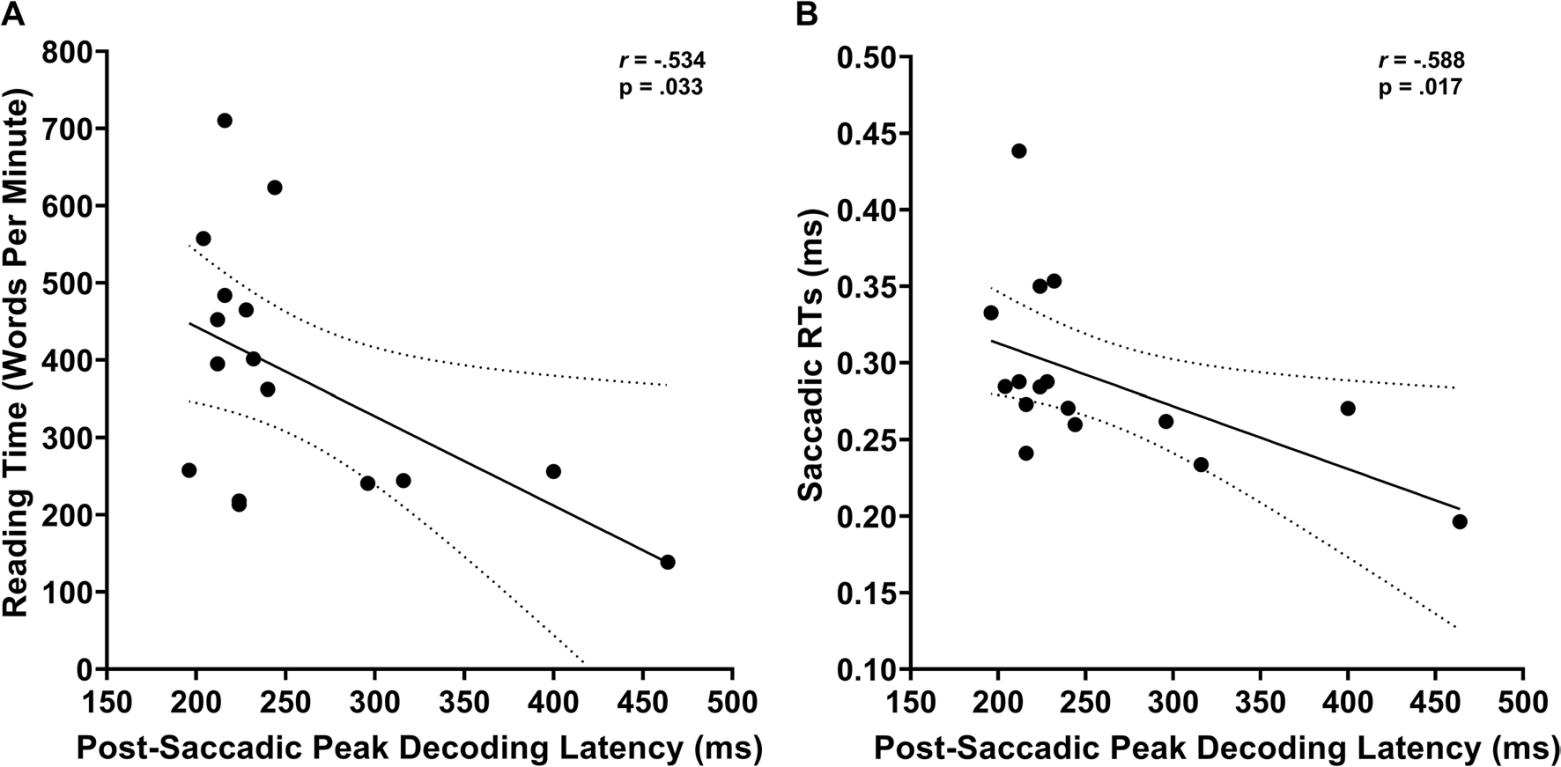
Significant Pearson correlations between post-saccadic condition and behavioral measures. (**A**) Negative association between decoding latency onset of post-saccadic condition and reading time (i.e., words per minute). (**B**) Negative association between decoding latency onset of post-saccadic condition and saccadic reaction times (ms). Black dots represent individual datapoint; shaded gray lines represent 95% confidence intervals

Finally, we considered the key parameters indexing the decoding of neural activity related to the post-saccadic stimulus condition (valid vs. invalid) and whether they were potentially linked to reading and oculomotor measures (Fig. 12). This analysis revealed that the latency of the decoding peak was negatively correlated with reading time (r = -0.534, *p *= 0.033) and the mean of saccadic RTs (r = 0.588, *p *= 0.017). Hence, these results revealed that the decoding latency dynamics associated to post-saccadic stimulus processing were informative regarding saccadic and reading temporal dynamics, suggesting that individuals who showed faster saccades and faster reading times also were those who encoded more efficiently post-saccadic stimulus information.

## Discussion

Visual perception typically involves looking at an object or word of interest after first choosing that saccade target using extrafoveal vision. In contrast, most EEG studies of object perception and word recognition have required participants to keep their eyes fixed, raising the question of how neural responses are influenced by the extrafoveal (typically parafoveal) preview. Here, we adapted a single-shot saccade paradigm from studies of object perception to investigate how the preview validity influences the evoked response to a word. Consistent with recent studies of word recognition, we found that the fixation-related response to the word, in particular at around 200–300 ms, was greatly reduced by a valid parafoveal preview of the word prior to the saccade. In addition, there was a second difference in the FRPs at around 400–500 ms, which was more focused in anterior electrodes compared to the earlier differences.

We used multivariate decoding analyses to further characterize these differences between valid and invalid preview trials, revealing several novel findings. We found early (< 100 ms) and sustained (up to almost 600 ms into the new fixation) decoding of the post-saccadic stimulus condition. Time generalization analyses revealed that, in contrast to decoding of the initial preview stimulus prior to the saccade, the classifier was able to decode across fairly disparate time points. This pattern is consistent with a relatively stable representation of stimulus category across the post-saccadic time period.

Interestingly, the strength of decoding was correlated with other aspects of individual participant performance. Of particular interest is the finding that participants who have a faster sentence reading speed, who also tend to rate English as their dominant language, show enhanced decoding of condition in the post-saccadic period. We use this multivariate decoding strength as a proxy for the strength of the influence of the preview on post-saccadic processing. As such, this suggests that people with a stronger processing of the words and a stronger preview effect for single words in this task are also faster readers more generally, in line with previous findings showing a link between preview effects and reading speed (Frömer et al., 2015; Pan et al., 2021; Risse, 2014). Interestingly, we find this link between preview effects and reading speed even when measuring the preview effect for single words.

The correlation analyses also provided evidence that participants who more strongly encoded the preview also showed a greater effect of that preview on post-saccadic stimulus processing. An interesting question for further research is what determines this difference in the “depth” of preview encoding between individuals. One possibility is that processing of the preview might vary from trial to trial depending on a general factor like attention allocation. Alternatively, some people might tend to process parafoveal information better due to idiosyncratic differences in the neural representations of parafoveal stimuli (Benson et al., 2021; Himmelberg et al., 2022), perhaps explaining the link between preview effect and reading speed. Additionally, there seems to be a strategic or experience-based component to preview effects as well. For the preview effect with faces, manipulating the preview validity across blocks also altered the strength of the preview effect. A stronger preview effect was found when the preview had always been valid in recent trials compared to when it had consistently been invalid (Huber-Huber & Melcher, 2021). Thus, past experience may play a role and some people may have learned to use parafoveal previews more than others, perhaps due to differences in the quality of parafoveal processing from person to person.

It is interesting to note that we found a strong effect of the parafoveal preview despite the fact that only half of the trials included valid cueing and that, in all trials, the fixated item was always a word. Given that the participants always saw a word after the saccade, this was a relatively weak violation of expectation or prediction in this experiment, at least compared to studies in which the category of the stimulus cannot be predicted in advance (de Lange et al., 2018). In most previous studies, preview effects and prediction/expectation effects have typically been confounded. The fact that we found a comparable preview effect in this reduced design, without a strong prediction violation (participants should have predicted seeing a word, not a number, after the saccade), suggests that making preview predictions may be somewhat automatic, and it may be possible to further distinguish between the roles of predictive and non-predictive aspects of the preview effect in future research.

The current study focused on recognition of a single word. Nonetheless, the magnitude of the preview effect found here with the single-saccade version was comparable to previous reports with word reading with word lists (Dimigen et al., 2012; Kornrumpf et al., 2016), as well as whole sentences (Burnsky, 2022; Loberg et al., 2019; Zhang et al., 2019). As in those reports, the fixation-related response for valid and invalid preview conditions was initially quite similar, in terms of an early positive P1 component in posterior electrodes at around 100 ms and then a negative deflection (N1 or N170) by around 150 ms. Consistent with those studies with word lists and sentence reading, we also found that the two conditions began to diverge at that point (around 200 ms), with the FRP in the valid preview condition returning back towards baseline, while the invalid preview condition continued to show a strong evoked response well into the 200- to 300-ms time period. Another difference between preview conditions was found approximately 400–500 ms into the new fixation. Previous studies have reported a preview effect on the N400 (Dimigen et al., 2012; Kornrumpf et al., 2016; Li et al., 2015; López-Peréz et al., 2016)). As in those studies, this difference in our data was more anterior than the late N1/N170 difference. This is interesting, because in earlier reports it was difficult to separate these late effects from foveal-load effects (for discussion, see Kornrumpf et al., 2016). In our study, there was no foveal load and only single words, suggesting that at least some of the differences in FRPs in those electrodes at that time reflect the nature of the preview stimulus itself. In the study by López-Peréz and colleagues (López-Peréz et al., 2016), they reported evidence for a late effect (N400) of the parafoveal preview, which overlapped into the new fixation, as well as parafoveal-to-foveal and foveal processing happening at the same time.

The current results can also be compared to studies of the flanker word effect (Eriksen & Eriksen, 1974; Shaffer & Laberge, 1979). Such studies, which present parafoveal flankers at the same time as the foveal word target, have shown that the parafoveal information can influence the RT to the foveal word stimulus (Dare & Shillcock, 2013). In that paradigm, unlike the current study, the parafoveal and foveal information are shown simultaneously, rather than sequentially. Snell and Grainger (2018) suggest that the presence of parafoveal flanker effects show that, in principle, parafoveal information can influence foveal processing. However, they suggest that the degree to which parafoveal information is integrated can depend on a myriad of factors that can all come to play depending on the task and the stimulus itself, such as different written scripts such as Roman-alphabetic versus Chinese characters. A similar argument could be made about the current results: we report that information about the parafoveal word can be decoded from the EEG signal early (< 100 ms) into the new fixation, interacting with the post-saccadic processing of the word. In more complex reading tasks, however, other factors may come into play related to the construction of sentence meaning, as well as differences that may arise from different scripts that lend themselves more or less to parafoveal processing. As such, the time course of decoding may differ in more complex reading tasks, perhaps revealing earlier or later effects not found in this simplified design. In addition, the use of only a single word is likely to have boosted decoding performance, since there were no competing word representations in the EEG signal from the previous or future word in the sequence.

Another interesting aspect of the current study is that the word was relatively small (1.5 dva) and presented isolated at a distance centered 3 dva from fixation. The magnitude of the preview effect, whether for a word or a face, is influenced by its visibility (Frömer et al., 2015; Liu et al., 2022). In the case of words, factors such as font size and visual crowding (determined by the relative spacing of the target and surrounding items) will influence the ability of the visual system to rapidly extract enough orthographic information to generate a useful preview (Yu et al., 2007). The single-shot saccade paradigm used here can be a tool in future work to isolate these bottom-up, visual effects and determine how they influence the preview effect in different languages and written scripts.

The use of the single-shot saccade paradigm allows for a more direct comparison with the visual trans-saccadic integration literature (see Introduction) and the preview effect for other types of visual stimuli, such as faces or gratings (for review, see Huber-Huber et al., 2021). In the case of faces, there is a reduction in the classic N170 face-related response, similar to the reduction in the N170 for words found here. With faces, there was evidence for an effect of preview stimulus type already in the EEG signal by 50–90 ms post-fixation onset, in at least two experiments (Huber-Huber et al., 2019). Given the similarity in the paradigms (with the exception of the face previews being further away from the fovea), it was interesting to now find early effects (< 100 ms) for words, as had been found for faces. It would be interesting in future work to more directly compare these preview effects in a within-subjects paradigm. In addition, the current paradigm allowed more time for the participant to preview the parafoveal word than would typically be the case in normal reading. Given that the parafoveal processing starts prior to the saccade and continues into the new fixation, it would be useful in future studies to see how the duration of the preview might influence the timing of the post-saccadic effects.

The current study focused on single word reading, limiting its ecological validity and relevance to understanding reading of sentences. Nonetheless, as argued above, word recognition is still a fundamental step in reading and so any process that influences word recognition could have later, knock-on effects on, for example, reading speed. Indeed, we show that the decoding measure of the preview effect and individual reading speed were correlated. This paradigm would seem best suited to studying, for example, how low-level visual properties such as font size, spacing, or crowding might influence the preview effect for single-word recognition. However, it is also worth pointing out that this paradigm could be enriched by adding additional context, such as by presenting one or more words visually (or with auditory stimulation) prior to the appearance of the parafoveal word. As shown by Kornrumpf and colleagues (Kornrumpf et al., 2016), preview effects can occur for words shown sequentially at the fovea. However, they found that the preview effect was stronger for natural (saccade) conditions than rapid serial presentation of stimuli on the fovea. Similarly, the preview effect for faces was stronger for an active saccade condition compared to when a stimulus changed retinal position in a similar way but without a saccade (Huber-Huber & Melcher, 2023). This suggests that paradigms, like this one, in which a saccade is included may be useful in understanding the preview effect. In future work, words could be, for example, presented sequentially at the fovea and then a parafoveal word could appear, allowing for precise control of the timing and size of the saccade while also adding richer context.

The current study used a particular type of invalid preview and mask, a number string. As described above, this choice was motivated by the aim of avoiding any potential effect of a violated, incorrect prediction. In boundary-paradigm studies in which the word is changed during the saccade, the post-saccadic evoked response is likely a combination of a lack of preview effect and a prediction violation response. In the current paradigm, the target was always a word. Thus, the invalid condition failed to provide any useful predictive information about the potential identity of the post-saccadic target. In future studies, the paradigm used here could be extended to explore an interesting range of preview masks. Such studies could reveal how the type of preview mask in the invalid condition can impact both the timing and the magnitude of these preview effects, and even to more closely test what kind of predictions are licensed by a parafoveal preview.

The current results add to the growing evidence that initial processing of the saccade target facilitates the rapid and efficient post-saccadic processing of the word on the fovea. The existence of preview effects has important theoretical and practical implications. In terms of theory, among other things, preview effects are relevant in terms of understanding how our brain is able to process two objects or words simultaneously, or at least in a temporally overlapping way. An efficient viewer processes both the foveal and parafoveal stimulus, although of course there is a longstanding debate where in the continuum of sequential-to-parallel this process lies (for reviews, see Andrews & Veldre, 2019; Jensen et al., 2021; Schotter et al., 2012). More generally, however, the mechanisms by which cognition coordinates parallel processing, multiplexing, or multitasking is of great importance. The brain must strategically solve multiple problems in a short amount of time, such as using past experience to make predictions in order to fine-tune current processing, to separate targets from distractors, to attend to both the external world and internal representations such as working memory or mental imagery, and so on. In previous work, we investigated the time course of extrafoveal visual processing using multivariate analyses to determine when information about the extrafoveal and the foveal stimulus was present in the brain before and after saccade onset, and when the pre-saccadic and post-saccadic information interacted (Fabius et al, 2020, 2023; Huber-Huber et al., 2019). Those results were consistent with foveal and extrafoveal representations being present in an overlapping way. In the case of word processing, the use of advanced methods such as frequency tagging with magnetoencephalography (MEG), for example, allows for a more precise estimate of the temporal overlap between foveal and parafoveal processing (Pan et al., 2021). As demonstrated in Pan and colleagues’ (Pan et al., 2021) MEG study, and in previous work (Frömer et al., 2015; Rayner, 1998; Risse, 2014), parafoveal processing occurs while foveal processing is still ongoing, but the amount of parafoveal processing varies depending on many features of the task and stimuli, and from person to person. Moreover, the processing of parafoveal information predicts reading speed (Pan et al., 2021). We confirmed this link between the parafoveal preview effect and reading speed, which suggests that understanding individual differences in the preview effect, and its underlying mechanisms, may provide a potential avenue for future interventions in dyslexia. There is evidence that dyslexia, and reading speed more generally, is related to temporal processing and specific aspects of the resting-state EEG signal, for example (Turri et al., 2023). In future work, it would be useful to investigate more closely the potential links between extrafoveal visual processing efficiency, EEG characteristics, reading speed, and dyslexia.

## Conclusion

We demonstrated that a valid parafoveal word preview influences the fixation-related response to that word upon foveating. This study confirms and extends recent research done with combined EEG and eye-tracking with visual object stimuli and with word lists and sentences. We also demonstrated that the influence of the preview on post-saccadic word processing might be earlier (< 100 ms) than previously suggested, potentially reconciling discrepancies and inconsistencies in the word and object recognition literatures. We found individual differences in the size of the preview effect, which was also related to behavioral, EEG, and oculomotor measures, posing further questions for future research.

## Data Availability

Data and are available via the Open Science Framework at https://osf.io/6rzba/

## Code availability

Analysis scripts are available via the Open Science Framework at https://osf.io/6rzba/
